# Factor-H-related protein 1 (FHR1), a promotor of para-inflammation in age-related macular degeneration

**DOI:** 10.1186/s12974-025-03499-z

**Published:** 2025-07-03

**Authors:** Andjela Sekulic, Sarah M. Herr, Kelly Mulfaul, Inga-Marie Pompös, Silvia Winkler, Carola Dietrich, Benedikt Obermayer, Robert F. Mullins, Thomas Conrad, Peter F. Zipfel, Florian Sennlaub, Christine Skerka, Olaf Strauß

**Affiliations:** 1 Experimental Ophthalmology, Charité - Universitätsmedizin Berlin, Corporate Member of Freie Universität Berlin and Humboldt-Universität zu Berlin, 10117 Berlin, Germany; 2 Institute of Cell Biochemistry, Hannover Medical School, 30625 Hannover, Germany; 3 Department of Neuroscience and Pharmacology, University of Iowa, Iowa City, USA; 4 Genomics Technology Platform, Max Delbrück Center for Molecular Medicine, Berlin, Germany; 5 Berlin Institute of Health at Charité, Berlin, Germany; 6 Core Unit Bioinformatics, Berlin Institute of Health at Charité - Universitätsmedizin Berlin, Berlin, Germany; 7 Institute for Vision Research, Department of Ophthalmology & Visual Sciences, University of Iowa, Iowa City, USA; 8 Faculty of Biosciences, Friedrich Schiller University, 07743 Jena, Germany; 9 Department of Infection Biology, Leibniz Institute for Natural Product Research and Infection Biology, 07745 Jena, Germany; 10 Sorbonne University, INSERM, CNRS, Institut de la Vision, 17 rue Moreau, Paris, F-75012 France

**Keywords:** Age-related macular degeneration, Retinal pigment epithelium, Complement system, Inflammation

## Abstract

Age-related macular degeneration (AMD), a multifactorial type of retinal degeneration represents the most common cause for blindness in elderly. Polymorphisms in complement factor-H increase, while absence of factor-H-related protein-1 (FHR1) decreases the AMD risk, currently explained by their opposing relationship. Here we identify a FHR1-driven pathway fostering chronic cellular inflammation. FHR1 accumulates below the retinal pigment epithelium (RPE) in AMD donor tissue and similarly the murine homolog, muFHR1 is abundant in three AMD-relevant mouse models. These mouse models express the muFHR1 receptor EGF-like module-containing mucin-like hormone receptor 1 (Emr1) on the RPE and on invading mononuclear phagocytes (MP), where both cells form clusters via muFHR1/Emr1. FHR1 ignited EMR2-dependent Ca^2+^-signals and gene expression in both human RPE cell line and in vivo where muFHR1 affects Emr1^+^ cells (RPE and MP) gene expression shown by RNAseq analysis. As muFHR1 deletion in mice revealed significantly reduced MP invasion and neoangiogenesis in laser-induced choroidal neovascularization, we hypothesize that FHR1 accumulates, stabilizes and activates MP in the stage of RPE degeneration.

**Supplementary Information:**

The online version contains supplementary material available at 10.1186/s12974-025-03499-z.

## Introduction

Age related macular degeneration (AMD) is a gradual loss of central vision as a result of a progressing damage of the outer retina accompanied by a chronic low-grade local inflammation. Hallmarks of AMD are loss of choroidal endothelial cells, retinal pigment epithelium (RPE) and photoreceptors [1]. Late stages of AMD are defined by geographic atrophy (dry AMD) or choroidal neovascularization (wet AMD) [2]. Despite being one of the major causes of vision loss in industrialized countries [3], available treatment is limited to manage dry AMD lesions by the new complement inhibitor: pegcetacoplan and avancincaptad [4]; wet AMD by inhibiting vessel formation with anti-VEGF therapy [2]. Inhibition of canonical complement activation proved lack of efficiency supporting a bigger likelihood for therapeutic approaches on non-canonical complement pathways.

A key aspect of AMD pathogenesis is RPE degeneration and the disruption of immune barrier of the retina. Physiologically RPE cells provide this barrier to modify and inhibit local immune responses [5, 6]. In the diseased, and/or aging retina, para-inflammation driven by cellular degeneration and pro-inflammatory cytokines overcomes the immunosuppressive capacity of the RPE, allowing immune cells to infiltrate the previously immune-privileged retinal space [7, 8].

The identification of complement deposits in drusen [9–12], as well as polymorphisms in genes coding for complement factors, suggest that altered regulation of complement activity is associated with the development of AMD [13, 14]. Dysregulation of the complement system and its systemic over-activity affect retinas functionally and phenotypically, particularly the first line of defense– the RPE. Lifelong exposure to components of the complement system induces differential RPE phenotype [10, 15–18].

A specific genetic variant (Y402H) in complement factor H (CFH) confers the highest risk of developing AMD later in life. Since the identification of CFH as a major susceptibility gene in 2005 [19–21], multiple replications and further genetic studies have been performed to investigate the involvement of complement regulators [22]. The CFH encoding gene is located in the *FACTOR H* gene cluster that includes CFH together with its splice variant FHL-1 and factor H-related genes (*CFHRs*) coding for the proteins FHR1-5.

Concerning FHRs, each FHR protein concentration was elevated in serum of AMD patients [23]. Those observations were further related to genetic variations. For example, frameshift variation in *CFHR5* reduced not only FHR5 serum levels, but also FHR2 and FHR4 [24] which may protect against AMD. While Cipriani et al. [25] correlated FHR4 levels with the protective allele of the strongest AMD-associated CFH locus variant, Zouache et al. [26] found these variants did not affect AMD susceptibility and were not independently associated with the disease. FHR3 levels remained unchanged in AMD [27], but local FHR3 was reported to shift the RPE immunogenic phenotype into a pro-inflammatory phenotype [28]. Furthermore, a common deletion of *CFHR3* and *CFHR1* is associated with the most protective haplotype [29] although their expression is restricted to the liver [29, 30]. Due to their high homology, FHR1-5 are believed to compete with CFH to bind C3b and thereby regulate the (canonical) complement system at different levels of the complement cascade to further aggravate AMD pathology [25, 31, 32].

One landmark paper demonstrated a non-canonical function of CFH that determined the binding of CD47^+^ mononuclear phagocytes (MP) to thrombospondin-1 to prevent their subsequent elimination [33]. Although supportive work by others substantiated the importance of Y402H CFH variant in non-canonical context [34–36], so far FHRs genetic variations were only regarded under canonical complement regulations in AMD pathology. However, a novel non-canonical mechanism of FHR1 has been discovered in vasculopathies [37]. Here, upon binding to necrotic sclerotic plaques, FHR1 activates the monocytic inflammasome via EGF-like module-containing mucin-like hormone receptor 2 (EMR2) and contributes to the sterile inflammation of atherosclerotic cardiovascular disease [37, 38].

In this study, we examine how FHR1 contributes to the development of AMD. We examine FHR1 in human AMD outer retina specimen and its mouse version, FHRE, in mice models relevant to AMD. This allows us to closely examine the damage to the immune barrier of the retina and the infiltration of MPs driven by FHR1. To avoid confusion about the identity of the protein (as FHRE sequence is also called FHRB in the database), we have changed the name of FHRE to murine FHR1 (muFHR1). In vitro experiments confirm a change of the RPE cell gene expression by FHR1. Our findings suggest that FHR1 fosters local cellular inflammation in the retina as a mechanism in AMD development.

## Materials and methods

### Human samples, staining and serum concentration

Human donor eyes were obtained from the Iowa Lions Eye Bank (Iowa City, IA, USA) in accordance with the Declaration of Helsinki and with full consent from the donor’s next of kin (Table S1). Immunohistochemistry was performed on paraformaldehyde (PFA)-fixed frozen cryosections from 3 AMD donors with geographic atrophy and 3 control donors with an antibody directed against FHR1. Tissue was blocked with 0.1% bovine serum albumin for 15 min followed by a 1-hour incubation with primary antibody against FHR1 (1:200 dilution). Tissue was washed and incubated with a 1:200 dilution of Alexa Fluor 647 donkey anti-mouse antibody (Invitrogen) in PBS with diamidino-2-phenylindole (DAPI) for 30 min. Tissue was washed and mounted with Aquamount and coverslipped. Tissue sections were imaged on an Olympus BX41 microscope.

As previously described [37], blood samples were collected from 89 patients from Charité Universitaetsmedizin Berlin diagnosed with AMD (60% female and 40% male) with a mean age of 78 and compared to the age-matched controls (in average, 63 years old, 62% male, 38% female). The data was anonymized. Blood samples were centrifuged with EDTA before storage of the serum at -80 °C. FHR1 concentration in human serum was determined using FHR1 (RayBiotech) Elisa Kit according to the manufacturing protocols.

### Animals

All experiments were conducted in accordance with the ARVO Statement for the Use of Animals in Ophthalmic and Vision Research. Mouse experiments performed in Berlin, using *Cx3Cr1*^*GFP/GFP*^, C57BL/6J (wild type, wt) and *muFHR1*^*−/−*^ animals were approved by the local authorities (Landesamt für Gesundheit und Soziales, LageSo, Berlin; Licenses– T-CH 0027/20 and G0005/21). Animals were maintained on a 12-hour light/dark cycle, under standard environmental conditions, and food and water were provided ad libitum. *Cx3Cr1*^*GFP/GFP*^ develop age-dependent subretinal inflammation, a hallmark of AMD, leading to hyperinflammation. Both young (2 months) and old (12 months) C57BL/6J were obtained from Charles River (Germany). We induced choroidal neovascularization in young (2 months) C57BL/6J mice, together with young *muFHR1*^*−/−*^ mice, as described previously[39, 40) and briefly below. *Cx3Cr1*^*GFP/GFP*^ have been investigated at different ages: 6 months (*n* = 1), 8 months *n* = 5) and 12 months (*n* = 10) to explore the age-dependent muFHR1 dynamics, while TRE2 animals were examined at age 12 moths. Age-matched C5BL/6J mice were used as controls for *Cx3Cr1*^*GFP/GFP*^ and *TRE2* animals. Both sexes have been used in equal amounts.

### *muFHR1*^−^^/^^−^ animals

muFHR1KO (*muFHR1*^*−/−*^*)* mouse was generated by the MAGEC laboratory (Walter and Eliza Hall Institute of Medical Research, 1G Royal Parade, Vic, 3052, Australia). Briefly, to create the ko the factor H-related gene 1 (fhr1) (NCBI accession nr NC_000067), was deleted by CRISPR-Cas9 technology in Mus musculus strain C57BL/6J chromosome 1, using 2 sgRNAs of the sequence CTCCATTCTGTAGTTACGTC and CAATGAGTATTGCATTAGGC. 20,823 bp of genomic sequence was targeted for deletion (position 139.488536) which is confirmed by Western blot analysis, RNA sequence analysis and staining of flatmounts. For detailed characterization please refer to Fig. S1.

### *TRE2* animals

*TRE2* mice were generated by targeted replacement engineering to express human APOE isoform, *TRE2*. The animal model has been previously described [33, 41, 42] and kindly provided by Dr. Patrick Sullivan. Mice have been backcrossed with C57BL/6 mice to eliminate the Crb1^rb8^. Mice were housed in the animal facility under specific pathogen-free condition, in a 12/12 h light/dark (100–500 lx) cycle with water and normal diet food available ad libitum. All experimental protocols and procedures were approved by the local animal care ethics committee “Comité d’éthique en expérimentation animale Charles Darwin” (Ce5/2010/013; Ce5/2011/033; Ce5/2010/044).

### Model of laser-induced CNV and fluorescence angiography

The model is based on RPE/choroid layer disruption using an argon laser [39, 40, 43]. For this purpose, three/four different laser spots (depending on the occurrence of the bleeding) were applied to the outer retina to induce neovascularization, as a model of choroidal neovascularization (CNV). Briefly, after animals were deeply anaesthetized with subcutaneous injection of ketamine (100 mg/kg) and xylazine (12 mg/kg) and pupils were dilated with phenylephrine tropicamide eye drops (Charité Pharmacy, Berlin, Germany), an argon laser (Visulas 532 s, Carl Zeiss Meditec, Oberkochen, Germany) set to 120 mW, 100 ms, and 50 μm perforated Bruch’s membrane without affecting major vessels. The occurrence of hemorrhage was recorded and the number of laser spots was adjusted accordingly. 14 days after laser treatment, newly formed CNV scars were evaluated by fundus angiography using a Spectralis HRA-OCT with a 55-degree lens (Heidelberg Engineering, Heidelberg, Germany) after injection of fluorescein (5 mg/kg, fluorescein 10%; Alcon, Freiburg, Germany). Leakage area and integrated density (IntDen) were quantified using ImageJ (1.53o, National Institutes of Health, Bethesda, MD, USA). Based on their characteristics, lesions were classified into different severity categories: no leakage, minimal leakage (< 2500 pixels), classic (round, controlled leakage), confluent (when at least two lesions would merge) and non-classic (uncontrolled leakage, usually due to bleeding during laser treatment). At the end of the in vivo experiments, the eyes were treated with Corneregel (Bausch & Lomb GmbH, Berlin, Germany) before sacrifice and enucleation.

### Immunohistochemistry in rpe/choroid flatmounts

After enucleation, the eyes were fixed in 4% PFA for 13 min. The cornea was dissected by a circular incision to remove the lens and vitreous. With four cuts from the peripheral fundus towards to the optic nerve, the inner eyecups were opened and flattened to allow removal of the retina after cutting the optic nerve. The remaining part of the flatmount, including RPE, choroid, and sclera were then permeabilized in 5% 100x Triton in TBS overnight at 4 °C. The samples were then blocked with 5% BSA solution for 5 h at 4 °C, followed by incubation overnight at 4 °C with primary antibodies for staining phalloidin (identifies RPE hexagonal structure), Iba1 (marker for mononuclear phagocytes), Emr1 (receptor for muFHR1) and muFHR1. Monoclonal antibody against muFHR1 was exclusively used to detect muFHR1. Samples were washed 3x for 5 min with TBS before secondary antibody incubation for 90 min at room temperature. Details of antibody selection and secondary antibodies are provided in (Table S2) Supplemental Material. Finally, the samples were washed again with TBS 3x for 5 min and mounted on glass slides with Mounting Fluorescence Medium (DAKO, Agilent Tech. Inc. Cat. No. S2023). Mounted samples were stored at 4 °C in the dark to avoid bleaching. Samples were examined with Leica SPE a confocal microscope (Leica Microsystems GmbH, Wetzlar, Germany) using Leica Application Suite X (3.7.4.23463; Leica Microsystems CMS GmbH).

### Cell culture

For Ca^2+^ imaging and gene expression experiments, ARPE-19 and iPSCs-derived RPE cells were used. ARPE-19 cells (ATCC, Cat. No. CRL-2302) were cultured in DMEM/F12 (Thermo Fisher, Cat. No. 11320033) supplemented with 10% FBS (Corning, Cat. No. 35-015-CF) and 1% penicillin/streptomycin (Bio&SELL, Cat. No. BS. A 2213) at 37 °C and 5% CO2. When necessary, ARPE-19 cells were dissociated with Accutase (BioLegends, Cat. No. 423 201) and plated on either 15 mm glass coverslips (Carl Roth, Cat. No. P232.1) or 6-well plates (Corning, Cat. No. 353 046) until they reached ideal confluence (~ 60% conflueney) to perform the desired experiment. We have opted to culture the cells in a semi-confluent state to mimic the pathological state of RPE cells during AMD development when RPE monolayer is damaged together with the immune-barrier. Furthermore, the preliminary data showed EMR2 expression localizes to the cell membrane in a semi confluent state (Fig. S2C). At least 5 different passages were used for each experimental setting including young (up to 10th passage), middle (10th -20th passage) and old (older than 20th passage). Prior to each experiment, non-confluent cells were maintained in DMEM-12 without FBS for 24 h.

The research team of Prof. Marius Ader (Center for Regenerative Therapies Dresden (CRTD), Technische Universität Dresden, Germany) kindly provided human RPE cells differentiated from induced pluripotent stem cells (iPSCs-derived RPE). iPSCs-derived RPE cells (differentiated from the iPSCs cell line CRTDi004-A; CTRD registered under https://hpscreg.eu/cell-line/CRTDi004-A) were grown on filter inserts in mTeSR™ plus medium (Stemcell Technologies, Cologne Germany) at 37°CC and 5% CO_2_ until they reached transepithelial resistance at 700 Ωcm2. Once the transepithelial resistance reached satisfactory level, iPSCs-derived RPE cells have been trypsinised for at least 15 min and further plated on 15 mm glass coverslips coated in Matrigel (Cat. No. CLS354230-1EA). The local Ethics Committee approved the use of human material under the registration number EA1/024/17.

### Calcium (Ca^2+^) imaging

Before each experiment, cells were maintained in serum-free medium for 24 h and then incubated with FURA-2/AM (2 µM, Merck, Cat. No. 344905) for 33 min at 37 °C. They were transferred to a custom-made recording chamber containing extracellular solution (in mM): 140 NaCl, 1.38 NaH_2_PO_4_, 5.21 NaHCO_3_, 0.6 MgCl_2_, 1.2 CaCl_2_, 7.25 KCL, 31.25 HEPES, 6 glucose and 0.51 mM MgSO_4_; and visualized with a Zeiss Axiovert 40 CFL inverted microscope (Carl Zeiss AG) equipped with a 40 × oil immersion objective, a Visichrome High Speed Polychromator System (Visitron Systems), and a high-resolution CCD camera (CoolSNAP EZ, Photometrics). The Fura-2/AM signal of intracellular Ca^2+^ was acquired using MetaFluor Fluorescence Ratio Imaging Software (Visitron Systems). The fluorescence intensity of Fura-2 was detected at an emission wavelength of 505 nm, while the excitation wavelengths were set at 340/380 nm. Changes in intracellular free Ca^2+^ upon application are presented as changes in the ratio in the fluorescence of the two excitation wavelengths (dF/F) relative to the baseline (ddF/F). In each experiment we applied either natural human serum (NHS) or FHR-deficient serum (ΔFHR1) 10% of the extracellular volume. To determine the FHR1 function, we have treated the cells with soluble (sFHR1) or immobilized (iFHR1) (10 µg/ml). Please note that iFHR1 was added in presence of either NHS/ ΔFHR1. To test FHR1/EMR2 interaction, ARPE-19 cells have been incubated with EMR2 antibody (10 µg/ml, R&D Systems, Cat. No. AF4894) for 2 h prior to the experiment.

### Gene expression analysis (Incubation, RNA isolation, cDNA transcription and RT-PCR)

Incubation: ARPE-19 cells were incubated with immobilized FHR1 (iFHR1) and NHS for 4 h at 37 °C and 5% CO_2_. To block EMR2, cells were previously incubated with an EMR2 antibody (10 µg/ml, R&D Systems, Cat. No. AF4894) for 2 h. RNA isolation: After incubation, RNA was isolated from ARPE-19 cells using the QIAGEN RNA Plus Mini Kit protocol (Qiagen Cat. No. 74136). Reverse transcription/qPCR: Isolated RNA was further transcribed into complementary DNA using Quantinova Reverse Transcription Kit (Qiagen Cat. No. 205313) to investigate gene expression and relative changes of IL1β and IL18 using SYBR Green (Biozym) Master Mix (Biozym Blue S’Green qPCR Kit, Cat. No. 331416 S) for RT-PCR (Qiagen Rotorgene Q/Series software). Primers were obtained from Eurofins Genomics (Ebersberg, Germany): IL1β– F: TCG CCA GTG AAA TGA TGG CT; R: TGG AAG GAG CAC TTC ATC TGT T; IL18– F: GCT TGA ATC TAA ATT ATC AGT C; R: CAA ATT GCA TCT TAT TAT CAT G; GAPDH– F: TCA ACG ACC ACT TTG TCA AGC TCA; R: GCT GGT GGT CCA GGG GTC TTA CT. Results were further analyzed using ΔCT method in which the gene expression of interest was first normalized to the housekeeping gene (GAPDH) before continuing to calculate the expression fold change in comparison to the control. In the presence of EMR2 blocker, after normalizing the data to the housekeeping gene and the control results, data was additionally normalized to the control containing the EMR2 blocker to limit the effect of EMR2 blocker.

### (mu)FHR1: identification, protein and antibody production, immobilization

Although murine and human FHR proteins differ, FHRE was initially identified as the homologue of human FHR1 based on evidence that FHRE, like human FHR1, consist of five short consensus repeats with similar homologies to Factor H, particularly in the dimerization domain and the C-terminal regions. FHRE and FHR1 share homologies, according to the sequence of the dimerization domain and the induction of the protein, as previously shown [38]. To be precise, FHRE, like human FHR1, consists of 5 short consensus repeats. Both FHRE and human FHR1 show similar homologies to SCR1 and SCR2 to the corresponding Factor H SCR6 and SCR7. Additionally, FHRE contains a dimerization motif similar to FHR1, FHR2 and FHR5 (Fig. S3). To avoid the confusion about the identity of the protein, we have changed FHRE to murine FHR1 (muFHR1), especially since the sequence of FHRE in the database is also called FHRB.

Recombinant FHR1 was expressed in *Pichia pastoris* and purified by nickel chelate affinity chromatography as previously described [38], where we have checked and observed that the His-Tag does not affect the functional activities of the protein. CFHR1 mAb JHD10 was generated by immunizing mice with purified CFHR1 fragments. The antibodies for the detection of human FHR1 have been published before [37, 38]. Polyclonal and monoclonal anti-muFHR1 antibodies have been generated in rats by DAVIDS Biotechnology. For selectivity of the antibody clones were selected against murine factor H and recombinant proteins FHRB and FHRC. The monoclonal antibody did not recognize proteins in the FHRE ko mouse IHC, confirming the deletion of FHRE in the ko mouse and the specificity of the monoclonal antibody. Furthermore, the antibody was tested in western blot analysis of liver cells derived from WT and *muFHR1*^*−/−*^ mice (see Figure S1 below).

To immobilize FHR1 from its soluble form, we chose to bind soluble FHR1 to magnetic COOH beads (Roti^®^-MagBeads COOH HP58). Protein coupling was performed according to the manufacturing instruction. In brief, the beads were activated for 15 min in freshly prepared EDC (40 mg/ml), NHS (40 mg/ml) solution diluted in MES (0.1 mM) to further bind FHR1 at the recommended concentration (50 µg protein/mg beads) in PBS. To block any unwanted binding and to enhance FHR1 binding, the FHR1-beads complex was incubated in Tris (0.1 mM) prior to storage. Shortly before the experiment, coupled beads were washed in pure alcohol and reactivated for incubation with cells.

### Western blot analysis

Whole cell extracts were obtained from cultured cells and from tissues using RIPA buffer containing 20 mM Tris, 150 mM NaCl, 1% NP-40, supplemented with a cocktail of protease inhibitors (Thermofisher, #88018). 20 µg of protein lysates were run on 10% SDS-polyacrylamide gels and transferred on 0.45 μm nitrocellulose membranes (Thermofisher, #IPVH00010). Membranes were incubated for 1 h at room temperature with a blocking solution containing 5% non-fat dry milk (w/v) resuspended in TBST 0.1% buffer (50mM Tris-HCl, 150 mM NaCl, pH 7.4, and 0.1% Tween-20) and subsequently incubated with primary antibodies at 4 °C overnight in a solution containing 1% BSA in TBST 0.1% buffer (Thermofisher; #BP9703-100). The following antibodies were used at the dilutions indicated: anti-muFHR1 (generated by DAVIDS Biotechnologies), and anti-GAPDH (R&D Systems, #AF5718) at 1:1000, anti-FHR1 (generated by DAVIDS Biotechnologies), anti-CFH (sc-166608-HRP, Santa Cruz), anti-EMR2 (ab75190, abcam) and anti-B actin (AC-15, Novus) at 1:3000. After antibody incubation, the membranes were washed three times in TBST 0.1% (v/v) and incubated at room temperature for 1 h with a 1:2000 dilution of anti-goat (Agilent, #P044901-2) or anti-mouse (Agilent, #P044701-2) or with anti-mouse (Invitrogen, A15981) IgG horseradish peroxidase-conjugated (BioRad) or with anti-mouse (Invitrogen, A15981) in TBST 0.1% containing 2% non-fat dry milk (w/v). After four additional washes in TBST 0.1% (v/v), immunoblots were developed, using an enhanced chemiluminescence kit (GE Healthcare, #RPN2108) on Fusion FX imaging system (Velber).

### Data analysis and presentation

GraphPad Prism 10 (GraphPad Software) was used for quantitative and statistical analysis and graphing. Where possible, individual values are presented with the mean ± standard error of the mean (SEM). In absence of individual values, mean ± standard deviation is presented. Each experiment has been replicated at least 5 independent times. After eliminating outliers, different conditions were compared with either t-test, Mann-Whitney test or Multiple Comparison test depending on normality. Person’s correlations were calculated between Iba1^+^ and muFHR1^+^ cells. OMERO and FPBioimage have been used were used for visualization and processing of immunofluorescence data [44], while video has been made my ClipChamp. In vivo images were processed with ImageJ. Figures have been created with either CorelDraw or BioRender.

### Single nuclei RNA-sequencing (snRNAseq)

After enucleation and removal of unneeded parts of the eye, as described above, the choroid containing RPE and sclera was immediately snap frozen in liquid nitrogen and stored at -80 °C prior to shipment to the sequencing facility. Snap frozen choroids were transferred to a 1,5 mL eppendorf tube placed on ice and crushed with 10–15 strokes of a plastic pestle in 500 µL of NP-40 lysis buffer (10mM Tris-HCL pH 7.4; 10mM NaCl; 3 mM MgCl2; 0.01% NP-40; 1mM DTT; Complete EDTA-free protease inhibitor; 2% BSA; 1 U/µl Protector RNase inhibitor). 750 µL of NP-40 buffer were then added and the sample was incubated for 5 min on ice with gentle pipette mixing after 2,5 min. The suspension was filtered through a 70 μm pre-separation strainer into a fresh 1,5 mL eppendorf tube. The filter was washed with 50 µl Wash buffer (PBS, 1% BSA, 1U/µl Takara RNAse inhibitor), and nuclei were centrifuged sedimented at 500 g for 5 min at 4 °C in a swinging rotor centrifuge. The supernatant was removed, leaving ~ 50 µL to preserve the nuclei pellet, and 1 mL of cold Wash buffer were added without mixing. After 5 min incubation, the pellet was resuspended and centrifuged at 500 g for 5 min at 4 °C. The supernatant was removed, leaving ~ 50 µL to preserve the nuclei pellet. The pellet was resuspended in 500 µL of cold Wash buffer (depending on the pellet size) and filtered through a 40 μm Flowmi cell strainer. 2 drops of PI were added and the sample was incubated on ice for 2 min. Nuclei were sorted with a 100 μm nozzle in an Eppendorf tube containing 100 µL Sort buffer (PBS, 5% BSA, 2.5 U/µl Takara RNAse inhibitor.

Droplet-based single-nuclei RNA-seq was performed using the 10x Genomics Chromium Single Cell 3′ Kit (v.3.1) following the manufacturer’s instructions. For single nuclei gel bead-in-emulsions (GEMs) generation, we aimed for a target output of 10,000 nuclei for each sample. The amplified cDNA and final libraries were evaluated on a 4200 Tapestation (Agilent Technologies) using the HS-D5000 and HS-D1000 High Sensitivity DNA kits (Agilent Technologies), respectively. snRNA-seq libraries were sequenced on an Illumina NovaSeq 6000 instrument.

### SnRNAseq data analysis (integration and dimensionality reduction)

Sequencing data was processed with CellRanger version 7.2.0 against the mouse reference genome (version mm10-2020-A). Raw count matrices were subjected to post-processing using CellBender (v0.3.0) [45] and then analyzed with Seurat (v4.1.1) [46] and RStudio (version 4.3.3), using cells with at least 250 genes, less than 10% mitochondrial content and less than 30000 UMIs. Doublets were removed using DoubletFinder (v2.0.3) [47]. Cells with high Rpe65 expression - identified as RPE [48, 49]– were further subclustered using subset(). Normalization was carried using NormalizeData(), 2000 features were selected with FindVariableFeatures(). The integrated data were arranged through RunUMAP() following principal component analysis (PCA) performed with RunPCA() and cell clustering was conducted with FindClusters(). Differential expression within all clusters and RPE subset was performed using DESeq2(50) pseudobulk data. To test differences in cell type composition mixed-effect binomial model has been used.

## Results

### FHR1 present in choroids of AMD patients

**Fig. 1:**
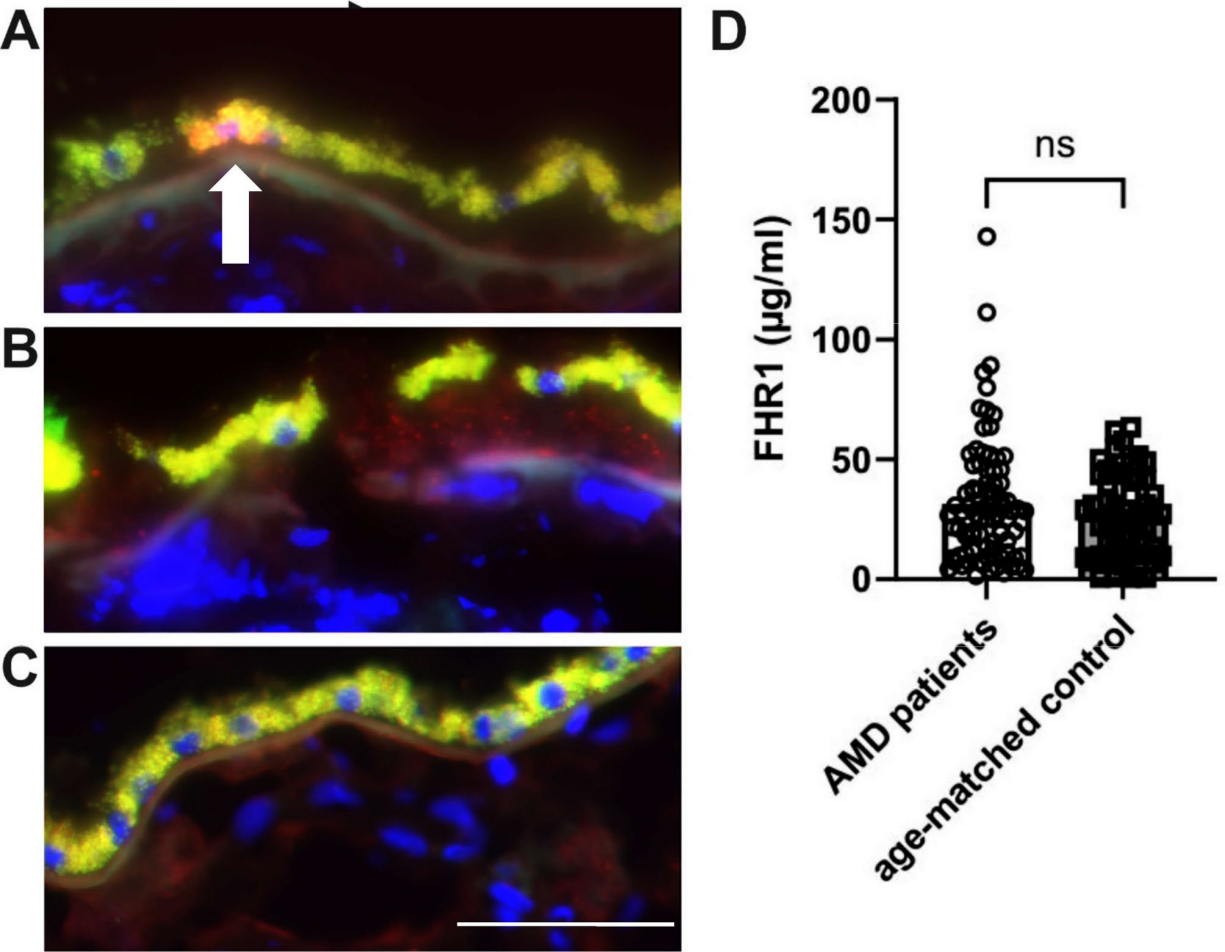
Labeling of human posterior pole with anti-FHR1 antibody. Immunolabeling against FHR1 (red) and DAPI (blue) in RPE/choroidal sections of AMD patients (*n* = 3) to localize FHR1 on the RPE (autofluorescent, yellow/green due to RPE lipofuscin) (**A**) and Bruch’s membrane/subRPE deposits (**B**) in the macula of a 96-year-old female donor with geographic atrophy. Modest labeling (**C**) is also observed in the macula of an unaffected 70-year-old male donor. Scalebar– 50 μm. FHR1 serum concentration between AMD patients (31.52 µg/ml *±* 2.73; *n* = 86) and age-matched controls (26.52 µg/ml *±* 2.34, *n* = 55). Statistical test performed by Mann-Whitney test, *p* = 0.5197, ns– not significant (**D**)

Natural deletion of *CFHR1/3* genes (5–7% in Caucasians [51] protects against AMD [29]. However, the mechanisms by which FHR1 potentially contributes to AMD-relevant patho-mechanisms remains elusive. To gain insight into an association of FHR1 in AMD pathology, we have labeled macular RPE-choroid sections of 3 donor eyes with geographic atrophy using an antibody directed against FHR1 and compared them to 3 age-matched controls. We observed RPE cells labeled with FHR1 in 2 of 3 AMD donors (Fig. 1A, white arrows). The RPE appears yellow/green due to autofluorescence, orange areas indicate areas of FHR1 labeling. In addition, we observed punctate labeling of FHR1 below the RPE and along Bruch’s membrane in the same AMD donors (Fig. 1B). We have observed similar FHR1 labelling in a second AMD donor, but due to the positive secondary control we have opted to demonstrate the findings in the supplement (Fig. S4). Third, unaffected AMD donor showed weak FHR1 staining demonstrating the variety of phenotypes and AMD severity (Fig. 1C). In contrast to AMD patients, 2 out of 3 control donors exhibited significantly lower FHR1 staining, and no evidence of punctate labeling below the RPE (Fig. S4). Interestingly, 3rd control donor exhibited strong FHR1 signal on RPE cells. While this donor may represent clinical control without a diagnosis, the choroidal samples shows signs for very early AMD (including drusen). A full panel of 3 AMD and 3 control donors is presented in (Fig. S4).

According to the presence of FHR1 basolateral to the RPE in AMD donors, the FHR1 serum concentration was determined in 86 AMD patients and 56 age-matched controls. The FHR1 concentration in the AMD patients’ serum was higher (31.52 µg/ml *±* 2.73) than that of the controls (26.52 µg/ml *±* 2.34) (Fig. 1D). To assess local expression of FHR1, western blot showed CFH protein expression in ARPE-19 cells (as expected(19)), but no FHR1 (Fig. S5A). Lack of FHR1 synthesis from RPE cells and other cell type has been confirmed with our snRNAseq (Fig. S5B) analysis and publicly available single cell RNA sequencing [52, 53]. The results suggest that while FHR1 levels differed slightly systemically, the protein does migrate locally from the choroid to the damaged sites in the retina.

### Accumulation of muFHR1 is confirmed in murine models of dry AMD over time

**Fig. 2:**
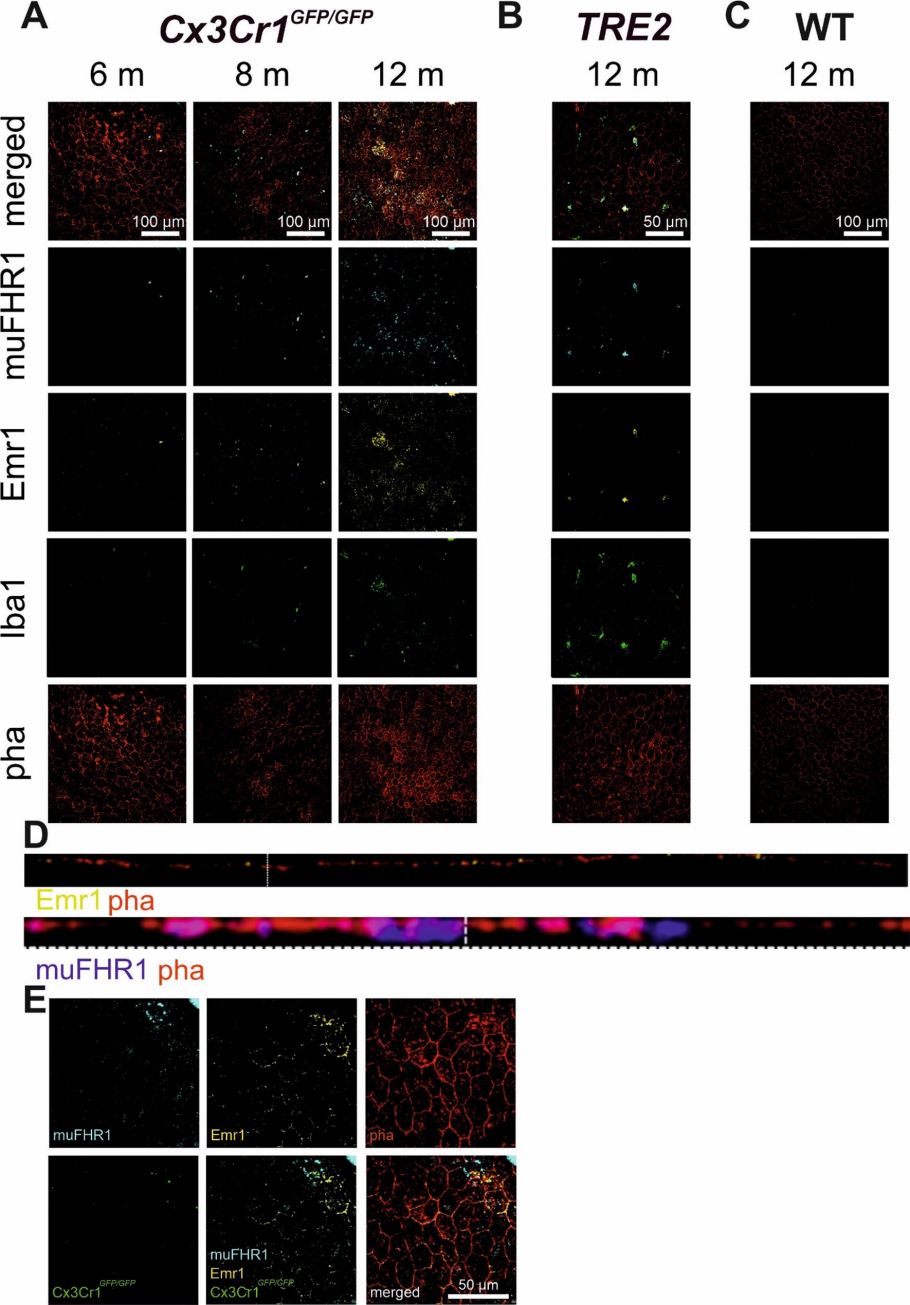
Gradual age-dependent muFHR1 accumulation in a mouse model relevant to dry AMD. muFHR1 (cyan), EMR1 (yellow), Iba1/Cx3Cr1 (green) and phalloidin (red) were detected in *Cx3Cr1*^*GFP/GFP*^ at different ages (**A**): 6 months (*n* = 1), 8 months (*n* = 4) and 12 months (*n* = 7); *TRE2* (*n* = 5) (**B**) and wild type (WT, C57B/6J) (*n* = 5) (**C**) mice at 12 months. Orthogonal plane to showcase the localization of Emr1 and muFHR1 in regards to phalloidin staining (**D**). muFHR1/Emr1 colocalization independent of Iba1 staining in 12 months old *Cx3Cr1*^*GFP/GFP*^ (**E**)

To ascertain the role of FHR1 accumulation in damaged areas of the retina of AMD patients, two mice models relevant to AMD exhibiting age-dependent properties of dry AMD: *Cx3Cr1*^*GFP/GFP*^ and *TRE2* were investigated. The *Cx3Cr1*^*GFP/GFP*^ animals develop low-grade chronic subretinal inflammation, a hallmark of AMD [54, 55], due to impaired activity control of mononuclear phagocytes (MP) and subsets of T cells. *TRE2* animals express elevated levels of human ApoE, a risk factor for AMD, which leads to subretinal MP accumulation and photoreceptor degeneration with age [56, 57]. RPE/choroid flatmounts were stained for murine FHR1 (muFHR1) and MPs. *Cx3Cr1*^*GFP/GFP*^ mice showed at the age of 6 months first signs of muFHR1 presence that increased after 8 months resulting in high muFHR1 build-up in the RPE at 12 months (Fig. 2A).

Irmscher et al.[38] demonstrated that immobilized FHR1 activates monocytes via EMR2. Consequently, the expression of the mouse homologue of human EMR2, Emr1, was examined in *Cx3Cr1*^*GFP/GFP*^ mice by immunostaining. The results demonstrate that Emr1 and Iba1^+^ cells (MP marker) increased in 12 months-old mice on the apical side of RPE that faces the photoreceptors (*Cx3Cr1*^*GFP/GFP*^: Fig. 2D). Similarly, we observe muFHR1 accumulation on the apical side of RPE cells in proximity to Emr1 (*Cx3Cr1*^*GFP/GFP*^: Fig. 2E). However, one should not exclude the possibility of muFHR1 binding to the Emr1 on the basolateral RPE membrane, as we observed FHR1 on the basolateral side of RPE in the human choroidal staining and preliminary examination of the Z-stack of stained mouse choroids (*Cx3Cr1*^*GFP/GFP*^: Fig. 2D).

**Fig. 3:**
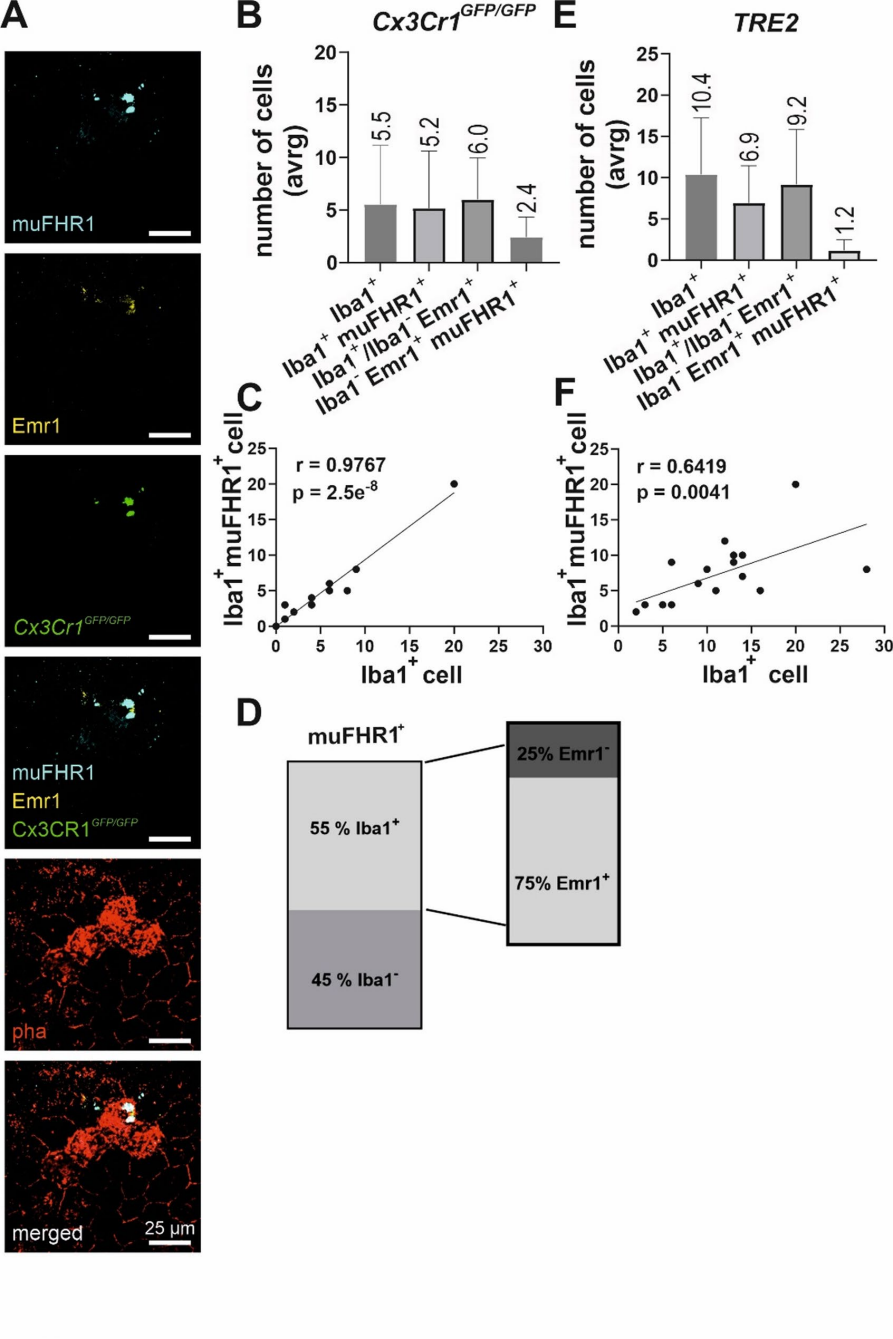
Interaction between muFHR1 and RPE-MP cells via Emr1. Interaction between RPE (immunolabeled with phalloidin– red) and mononuclear phagocytes (MP) (genetically engineered to fluoresce green) mediated through muFHR1-Emr1 (cyan blue-yellow) complex in Cx3Cr1^GFP/GFP^ flatmount choroid (**A**). Counted cells per imaged Cx3Cr1^GFP/GFP^/TRE2 sample and their differential expression profile (**B/E**). Pearson’s correlation of counted MPs expressing muFHR1^+^ in both Cx3Cr1^GFP/GFP^ (*r* = 0.9767, ****p < 0.0001) (**C**) and TRE2 (*r* = 0.6419, **p = 0.0041) (**F**). Heterogenous cell expression in the Cx3Cr1^GFP/GFP^ choroid (**D**)

A more thorough examination of muFHR1/Emr1 staining revealed colocalization of muFHR1/Emr1 with RPE and MP (Iba1^+^ cells) (*Cx3Cr1*^*GFP/GFP*^: Fig. 3A) suggesting complex formation. Therefore, we performed a quantitative analysis of the muFHR1/Emr1 staining on Iba1^+^ and Iba1^−^ cells. muFHR1 and Emr1 were found on Iba1^+^ cells in both mouse models but with substantially lower expression on Iba1^−^ cells (*Cx3Cr1*^*GFP/GFP*^: Fig. 3B; *TRE2*: Fig. 3E). Thus, it can be inferred that muFHR1 binds via Emr1 to both RPE cells and MP. The mean number of Iba1^+^ cells per imaged choroidal area was 5.5 *±* 1.7, with the majority being muFHR1^+^. Annotating the muFHR1^+^ (*Cx3Cr1*^*GFP/GFP*^: Fig. 3D), the majority represents MP (55%) compared to other cells (e.g. RPE, 45%). Not all Iba1^+^ cells expressed Emr1 (25%), despite the presence of muFHR1 positivity, suggesting the existence of a second muFHR1 binding receptor on these cells. A significant correlation between muFHR1 and Iba1^+^ cells was confirmed thereby associating muFHR1 with invading MPs (*Cx3Cr1*^*GFP/GFP*^: Fig. 3C).

We observed analogous kinetics in the second mouse model relevant to dry AMD, *TRE2*. Mice aged 12 months exhibited robust muFHR1 staining of both RPEs and MPs (*TRE2*: Fig. 2B) in comparison to their age-matched C57BL/6 control mice (Fig. 2C). Once more, muFHR1 signals depended on the cell type. The average number of Iba1^+^ cells (10.4 *±* 1.6), also exhibited muFHR1 staining (6.9 *±* 1.1) (*TRE2*: Fig. 3E). A small number of muFHR1^+^ cells were also Iba1^−^ (1.2 *±* 1.3) (*TRE2*: Fig. 3E). A notable finding was the significant correlation between muFHR1^+^ signals and the presence of Iba1^+^ MP in *TRE2* mice (*TRE2*: Fig. 3F). Collectively these findings demonstrate that in both models relevant to dry AMD the majority of the infiltrating MPs stain positive for muFHR1 and Emr1, indicating an interaction between the plasma protein muFHR1 and Emr1 which is expressed on MPs. Similarly, RPE cells express Emr1 to bind muFHR1 facilitating the interaction between RPE and MPs.

### muFHR1 binds both damaged and stressed RPE and MPs in CNV mouse model

**Fig. 4:**
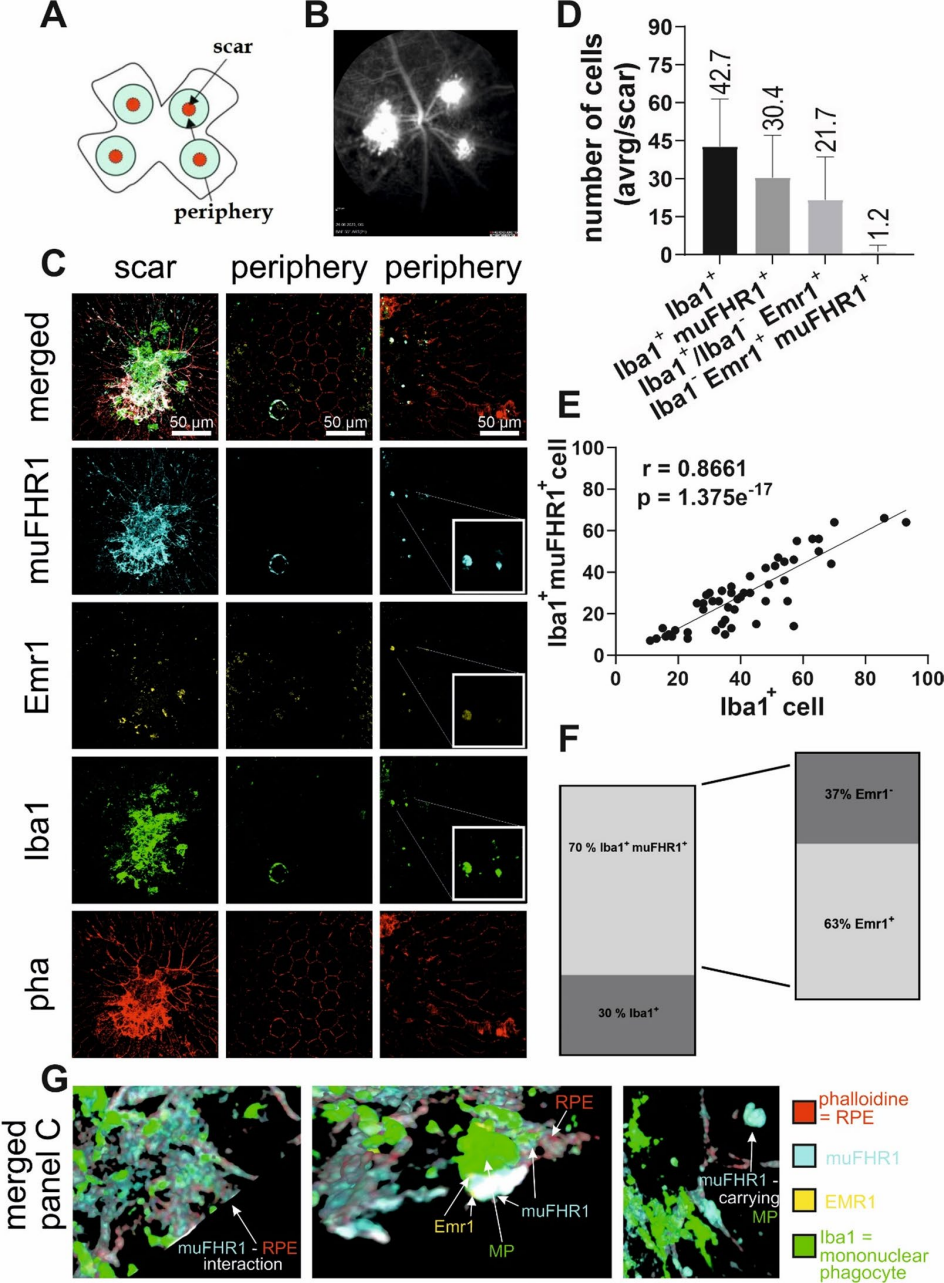
muFHR1 accumulates in an acute lesion of choroidal neovascularization (CNV). Flatmount preparation 14 days after laser-induced CNV in the mouse eye (*n* = 14). Schematic representation of flatmounted RPE/choroid showing the arrangement of laser burns, perilesion area (spot) and periphery (surround) (**A**). In vivo imaging of fluorescein leakage to show newly pathologically formed vessels (**B**). Immunofluorescence staining of muFHR1 (cyan blue), Emr1 (yellow), mononuclear phagocytes - MP (Iba1 in green) and RPE (phalloidin in red) 14 days after the laser burn at the injured spot and periphery (**C**). Quantification of cells that expressing the following signals: Iba1 and/or muFHR1 and/or Emr1 (**D**). Strong correlation was observed between Iba1^+^ and muFHR1^+^ Iba1^+^ cells (Pearson’s correlation, *r* = 0.8661, ****p < 0.001) (**E**). Annotation of quantified cells and their ratio based on expression profile (**F**). Detailed observation of the 3D merged panel C showing the: (**a**) muFHR1-RPE interaction; (**b**) RPE-MP interaction via muFHR1-Emr1; (**c**) muFHR1-bearing MP (**G**)

MPs, RPEs and muFHR1 colocalize in two AMD mouse models with characteristics for dry AMD. Thus, we investigated muFHR1 in a mouse model of choroidal neovascularization (CNV) [39]. In this model, laser-induced rupture of RPE and Bruch’s membrane (Fig. 4A) leads to endothelial proliferation, immune cell infiltration and a robust inflammatory response [43]. The phenotype was examined over the period of 14 days after the laser treatment - a time point at which neovascularization and scar formation reach their full phenotype. CNV was investigated in vivo by fluorescence angiography (FA) (Fig. 4B), to quantify the formation of new unphysiological blood vessels. The size of the vessels was measured by the area of fluorescein leakage, while the intensity of the leakage was gauged by the integrated fluorescence density. Subsequently eyes were enucleated and subjected for immunohistochemistry of the RPE/choroid complex to visualize muFHR1-Emr1 occurrence in conjunction with MP invasion.

**Fig. 5:**
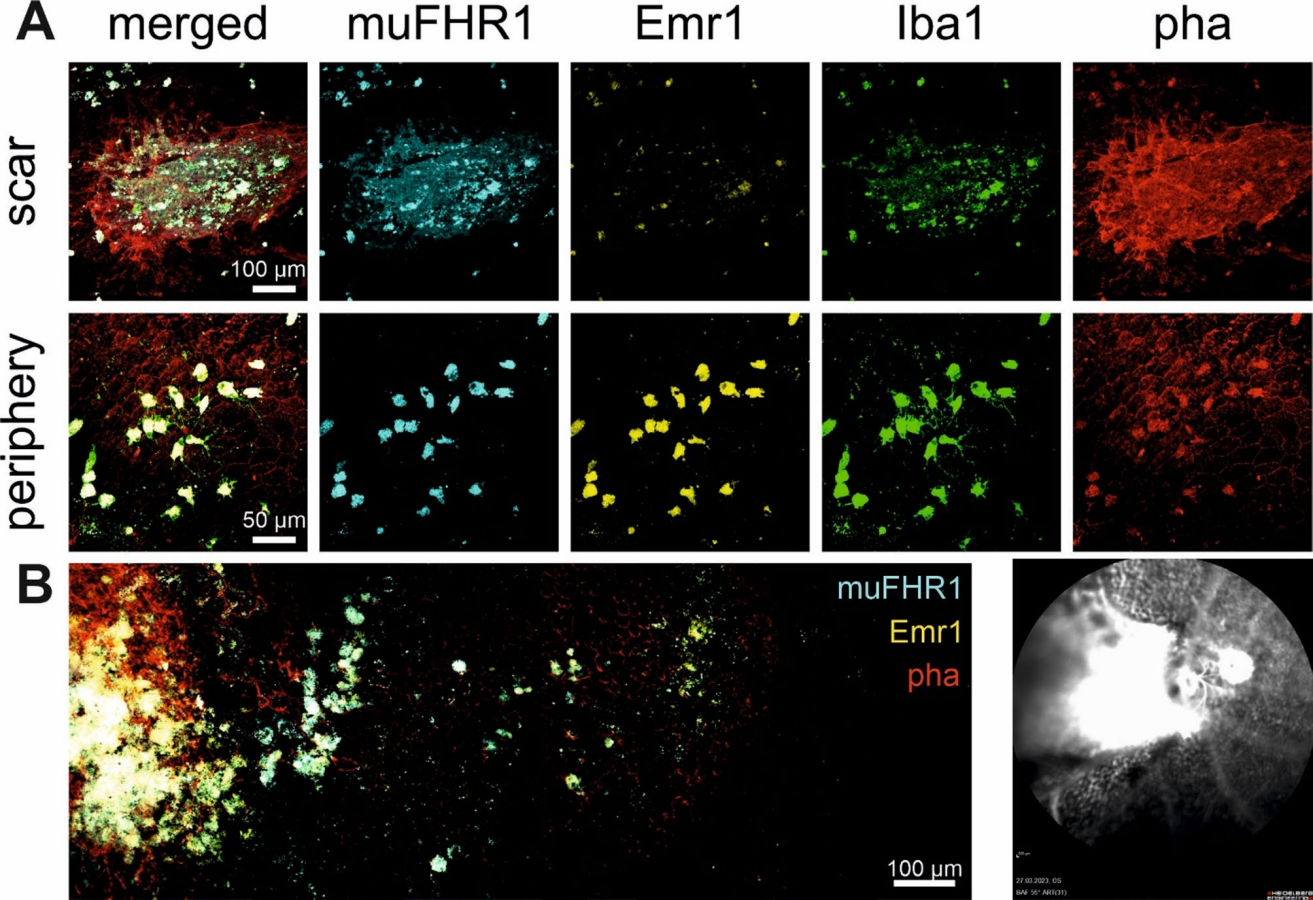
The dynamics of muFHR1 expression is determined by the spread of MP and serum. Stronger and further muFHR1(cyan) and Emr1(yellow) effect in case of hemorrhage upon laser ablation (*n* = 6) within the lesion and in the periphery followed by stronger invasion of mononuclear phagocytes - MP (Iba1^+^) cell(green) (**A**). Consecutive imaging of neighboring regions starting from the lesion visualizes the culmination of muFHR1 (cyan blue), Emr1 (yellow) and MP (green) and the ability of the protein-receptor-immune cells to migrate to the periphery (**B**). Visualization can be achieved with both immunofluorescence and in vivo angiofluorescence (**B**)

Already 4 days after the lasering, muFHR1 accumulated at the laser lesion sites (Fig. S6), but 14 days after, a robust muFHR1 signal persisted and showed the protein binding to both damaged RPE and infiltrating MP (Fig. 4C). The muFHR1 signal was accompanied by the Emr1 (Fig. 4C) signal exhibiting a strong correlation between muFHR1 and MP (Fig. 4E). The quantification of Iba1^+^ cells within each lesion revealed a mean number of 42.7 *±* 2.5 with 30.4 *±* 2.2 cells of being muFHR1^+^ (Fig. 4D). However, the signals were substantially reduced in Iba^−^ cells, as only 21.7 *±* 2.8 were counted to be Emr1^+^ and at least 1.2 *±* 0.3 cells that expressed both muFHR1 and Emr1 but without Iba1^+^ (Fig. 4D). In the periphery, infiltrated MPs also stained for muFHR1 and Emr1 (Fig. 4C) reaching out for RPE cells more distant from the scar (Figs. 4F and 5B). Interestingly, peripheral RPE cells not only expressed Emr1– stress indicator– but also bound muFHR1, despite appearing structurally intact (Fig. 4C) enabling the variability within cell types.

In order to comprehend the interactions between RPE and MP via muFHR1-Emr1 complex, we have utilized First Person BioImage– 3D feature to process volumetric data of the image analysis program OMERO [58]. Video 1 (S1) confirmed the following: (a) muFHR1 binds to the RPE damaged choroid; (b) interaction between RPE-MP is directed via muFHR1-Emr1 complex formation and (c) MP carry muFHR1 into the compromised choroid.

Analogous to the diversity between patients, we showed a variety of scar phenotypes, even if there is a bleeding upon lasering (Fig. 5A). In case of bleeding, the lesion phenotype appeared larger and muFHR1 distributing over larger area of the flatmounts accompanied with enhanced and strongly ramified autofluorescence MPs which have intruded the scar and beyond (Fig. 5A). Notably, muFHR1 staining patterns appeared (Fig. 5B) as expansion that followed the reach of the bleeding, supporting blood as the source of muFHR1 transporting MPs.

Our histological analysis with localization of Emr1 in apical and basolateral membrane of degenerating RPE cells, the binding to muFHR1 and MPs suggest cellular inflammation promoting function of FHR1 in AMD.

### FHR1 promotes an inflammatory phenotype in human RPE cells via EMR2

**Fig. 6:**
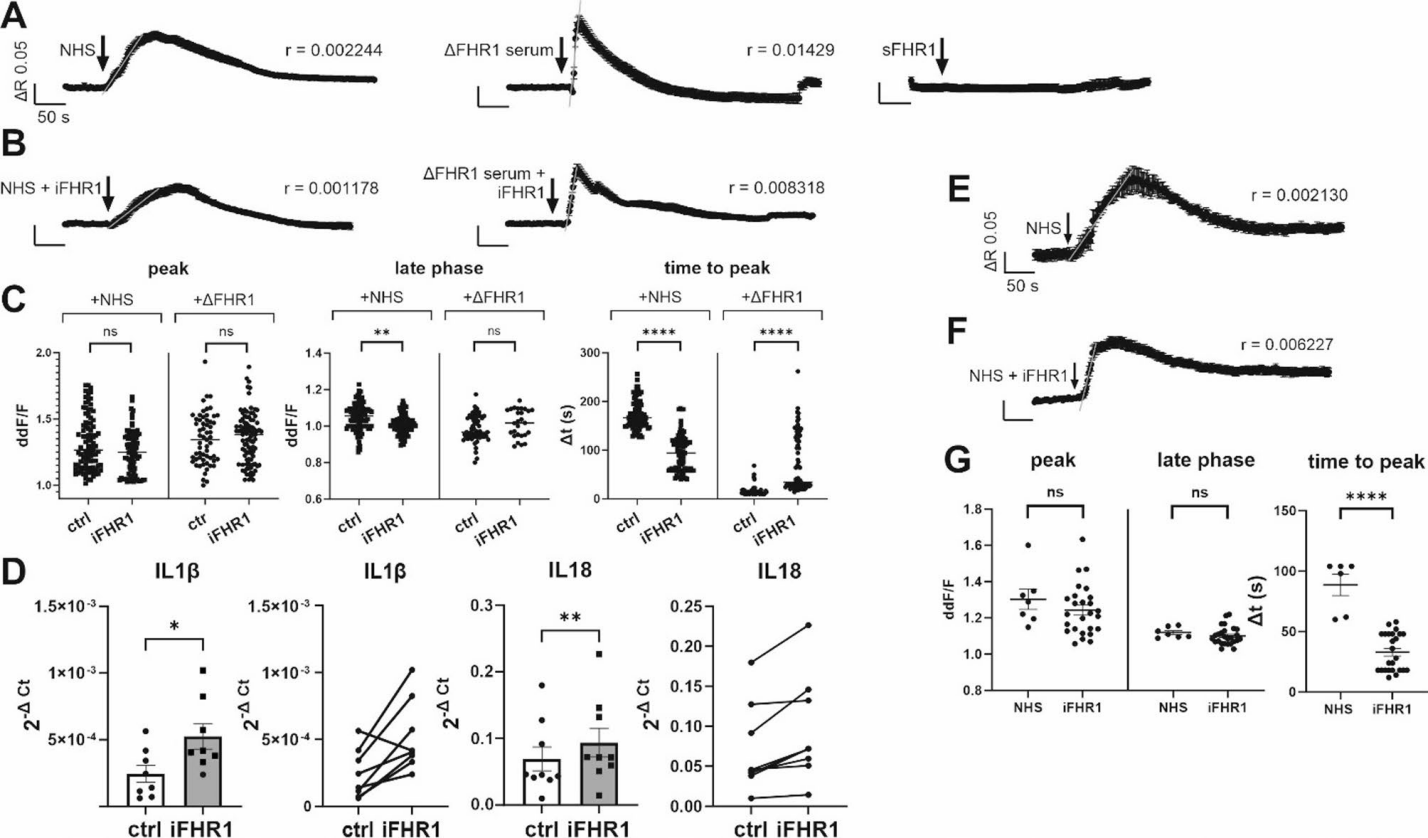
Immobilized FHR1 (iFHR1) induces inflammatory cytokine expression in ARPE-19 and iPSCs-derived RPE cells. Intracellular free Ca^2+^ was measured in non-confluent ARPE-19 cells using the Ca^2+^ -sensitive fluorescent dye Fura-2; changes in intracellular Ca^2+^ were plotted as changes in the fluorescence ratio of the excitation wavelengths. Longitudinal mean + standard error of the mean (SEM) of Ca^2+^ influx in ARPE-19 cells after application of 10% natural human serum (NHS, *n* = 93) or ΔFHR1 serum (*n* = 68) or soluble FHR1 (sFHR1, *n* = 36) (**A**) or in the presence of iFHR1 (10 µg/ml, *n* = 87/96) (**B**). Comparison of iFHR1-evoked Ca^2+^ signal at its’ peak, late phase and time to peak (multiple comparison Kruskal-Wallis test, **p = 0.0021, ****p < 0.0001) (**C**). Changes in IL1β and IL18 gene expression after 4 h iFHR1 incubation + NHS (IL1β– Wilcoxon test, *p = 0.0234; IL18– paired t-test, **p = 0.0042, *n* = 8) (**D**). Ca^2+^ influx in iPSCs-derived RPE cells induced by NHS (**E**) or + iFHR1 (**F**). Comparison of iFHR1-evoked Ca^2+^ signal within its’ peak, late phase and time to peak (Šídák’s multiple comparisons test, ns *p* ≅ 0.94, ****p < 0.0001) (**G**)

Bound FHR1 recruits and activates monocytes via EMR2 [38]. We found that not only MPs did express Emr1, but RPE cells were also susceptible to expressing Emr1 in stressed/diseased conditions. Additionally, we observed EMR2 protein expression in ARPE-19 cells (Fig. S2). These observations imply an intracellular signal transduction pathway that activates upon FHR1 binding to EMR2. We then tracked changes in intracellular free Ca^2+^ as a second-messenger using Ca^2+^-sensitive fluorescence dye (fura-2). According to the central dogma of intracellular Ca^2+^ signaling, the waveforms of Ca^2+^ elevation (specific in amplitude and kinetic) trigger specific changes in cell function [59]. Because the FHR1/EMR2 depends on various conditions and factors (such as natural human serum (NHS) and immobilizing beads), it is not possible to demonstrate a direct effect of FHR1/EMR2 binding. Its’ signaling depends on immobilization of FHR1 and unknown factors from the NHS bearing the possibility that non-FHR1 components trigger Ca^2+^ response as well. Thus, to extract FHR1-dependent signaling, we compared the waveforms under different conditions (Fig. 6A). As a control, isolated soluble FHR1 (sFHR1) did not cause a Ca^2+^ response, which is in line with already published data [38].

When we used immobilized FHR1 (iFHR1), together with either natural human serum (NHS) or ΔFHR1 (FHR1 deficient) serum we saw clear differences compared to the serum alone. The waveform in either iFHR1/NHS or iFHR1/ΔFHR1-serum changes the slope of the Ca^2+^ rise differently to either NHS or ΔFHR1-serum alone; indicating iFHR1 has a distinct effect (Fig. 6B-C). To understand if the waveforms elicited by iFHR1 actually trigger specific cellular activities, we investigated the IL1β and IL18 mRNA expression levels. Both increased after 4 h of iFHR1 incubation with NHS (Fig. 6D).

Despite being a common cell line in eye research, ARPE-19 cells considerably differ (in both phenotype and gene transcription) from native RPE cells [60]. Therefore, we repeated key experiments using RPE cells derived from induced pluripotent stem cells (Fig. 6E-F). The slope variation remained, and the cells reached their highest peak amplitude level faster than the ARPE-19 cells (Fig. 6G). However, the late phase shows differences between ARPE-19 and iPSC-RPE Ca^2+^ reaction. This could be explained due to the greater number of experiments conducted with ARPE-19 cells. As we suspect that the FHR1 would have the strongest effect in the early phases of the Ca^2+^ signal, we did not consider the discrepancy to be significant, as it probably indicates low biological relevance.

**Fig. 7:**
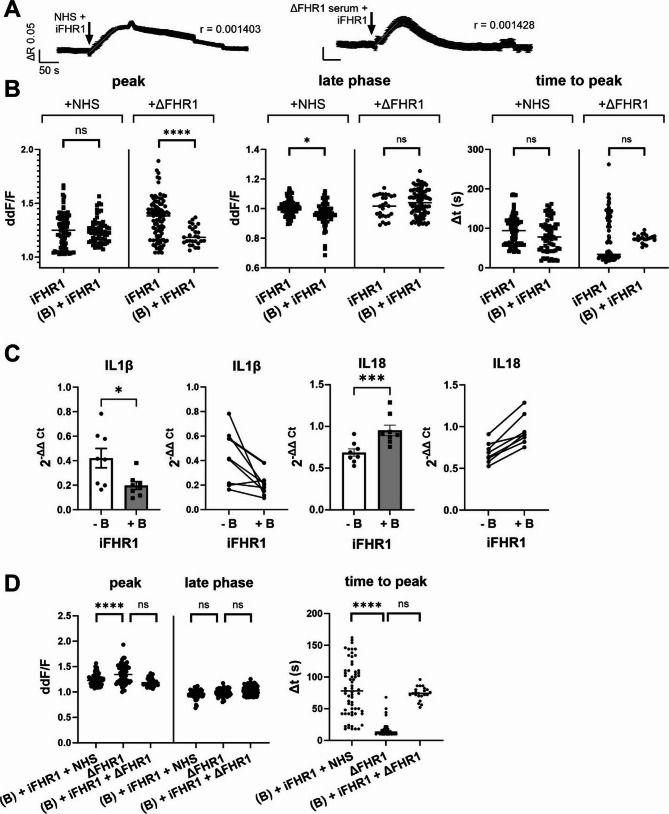
iFHR1 alters the expression of ARPE-19 cells via EMR2. EMR2 was blocked prior to incubation with iFHR1 + natural human serum (NHS) /ΔFHR1 serum to observe changes in both Ca^2+^ signaling and gene expression. Representative mean data + standard error of mean (SEM) of single experiments together acutely activated with NHS/ ΔFHR1 serum + iFHR1 (*n* = 63/27) (**A**). Comparison of response peak, late phase and time to peak after application (Kruskal-Wallis multiple comparison test, *p = 0.015, ****p < 0.0001) (Šídák’s multiple comparisons test, nonsignificant (ns) *p* ≅ 0.94, ****p < 0.0001) (**B**). Gene expression changes after 4 h incubation with iFHR1 + NHS in presence of EMR2 inhibitor (paired t-test, *p = 0.0324, ***p = 0.002, *n* = 8) (**C**). Comparison of Ca2+ signal peaks, late phase and time to reach the peak without EMR1 activation either due to presence of EMR2 blocker or due to absence of iFHR1 in serum (**D**)

To confirm that the increase in IL1β and IL18 mRNA expression levels stems from iFHR1-EMR2 interaction, EMR2 was subsequently blocked with an adequate antibody two hours prior to the iFHR1/NHS incubation (Fig. 7A). The Ca^2+^ influx was slower in the presence of either serum (iFHR1/NHS or iFHR1/ΔFHR1 serum) with the EMR2 blocker. The iFHR1/NHS Ca^2+^ reaction showed a lower later phase compared to the iFHR1/ΔFHR1 Ca^2+^ response which also revealed a decreased peak (Fig. 7B).

Along Ca^2+^ imaging experiments, we also performed gene expression analysis. As expected, when EMR2 was inhibited, FHR1 could not activate the receptor and downstream cascade, leading to a decrease in IL1β expression (Fig. 7C). On the other hand, IL18 expression increased after EMR2 was blocked (Fig. 7C). The specificity of blocker was validated by comparing the Ca^2+^ reaction amplitudes and kinetics in the absence or presence of FHR1 with the blocker (Fig. 7D). For a better understanding of the quantified Ca^2+^ response and the peak within the signal, please refer to the table in the supplement (Table S3). These results confirm that iFHR1, but not sFHR1 elicits a response and increases the gene expression of IL1β and IL18 via EMR2 binding using Ca^2+^ as a second messenger.

### Absence of muFHR1 ameliorates the severity of CNV

**Fig. 8:**
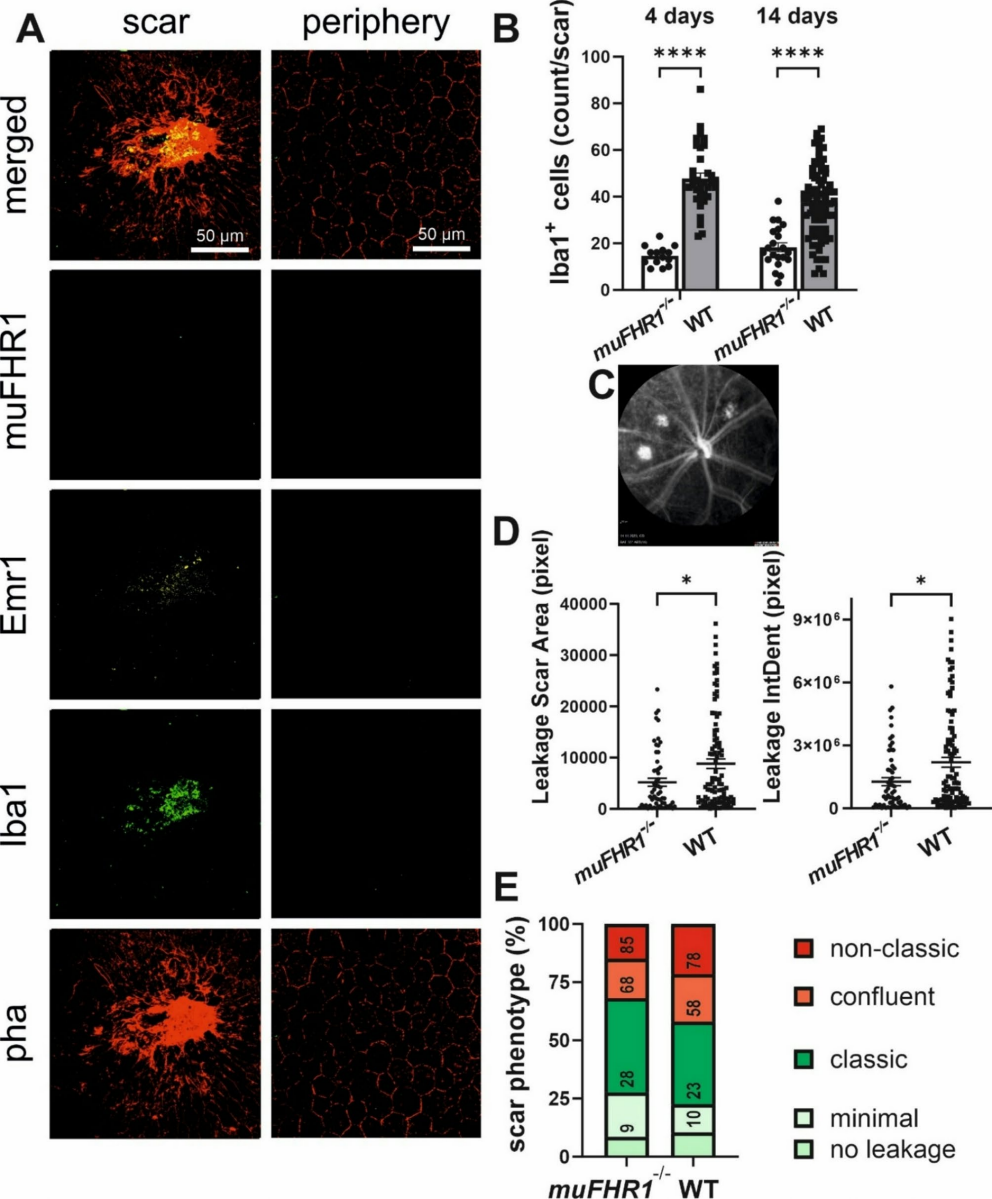
Deletion of muFHR1 ameliorates the development of induced choroidal neovascularization. Immunofluorescence visualization of the choroid in *muFHR1*^*-/-*^ mice 14 days after laser injury observed in destructed RPE (phalloidin, red) stained for no muFHR1 (cyan), low Emr1 (yellow) and invading MPs (Iba1, green) in both the laser spot and periphery (**A**). Invading MPs were significantly decreased at 14 days (unpaired t test, ****p < 0.0001, *n* = 7) (**B**) and 4 days (unpaired t test, ****p < 0.0001, *n* = 4) (**C**). In vivo fluorescence angiography (**D**) allowed comparison between leakage area and integrated density (IntDent) (Mann-Whitney test, *p = 0.0183) (**E**) and classification of lesion phenotype (**F**): no leakage– no fluorescein leakage where lesion is expected; minimal– leakage smaller than 2500 pixels; classic– round shape leakage with > 2500 pixels size; confluent– when two leakages merge into each other so they could not be distinguished; non-classic: where leakage is without clearly defined borders, usually after bleeding at laser

For demonstrating directly that FHR1 mediates cellular inflammation in the outer retina, we studied the model of laser-induced CNV paradigm in *muFHR1*^*−/−*^ mice and compared its symptoms with that in wildtype mice. When muFHR1 was absent there was a significant decrease in Emr1 signals within and around the scar area (Fig. 8A). Additionally, the peripheral RPE showed no Emr1 expression, and thus no signs of stress. Importantly, the number of Iba1^+^ MP significantly decreased by more than 50% in the laser areas of *muFHR1*^*−/−*^ mouse 4 days after the laser (the peak of immune activation; 14.64 *±* 1.1) and 14 days after the laser (18.29 *±* 1.93) (Fig. 8B).

We and others have previously reported that a reduction in numbers of the invading MPs coincides with reduced neovascularization and scar formation [39, 40, 61–65]. We then asked whether the changes in the scars’ phenotype depend on their genotype using FA (Fig. 8C). 14 days after choroidal laser damage, the area of fluorescein in *muFHR1*^−/−^ animals was significantly smaller compared to that in wild type mice (Fig. 8D). Additionally, significantly less fluorescein leakage, was seen in *muFHR1*^*−/−*^ mice (Fig. 8D). Evaluating the severity of laser-induced CNV formation by classification of scars into different categories (as a grading system) confirmed that *muFHR1*^−/−^ mice developed fewer non-classical or confluent scars and more classical, minimal lesions and even those that appeared with no leakage at all compared to wild-type mice (Fig. 8E). Taken together, the data show that muFHR1’s is directly involved in attracting MPs to the degenerating retina.

### muFHR1 affects all cell types involved in CNV

**Fig. 9:**
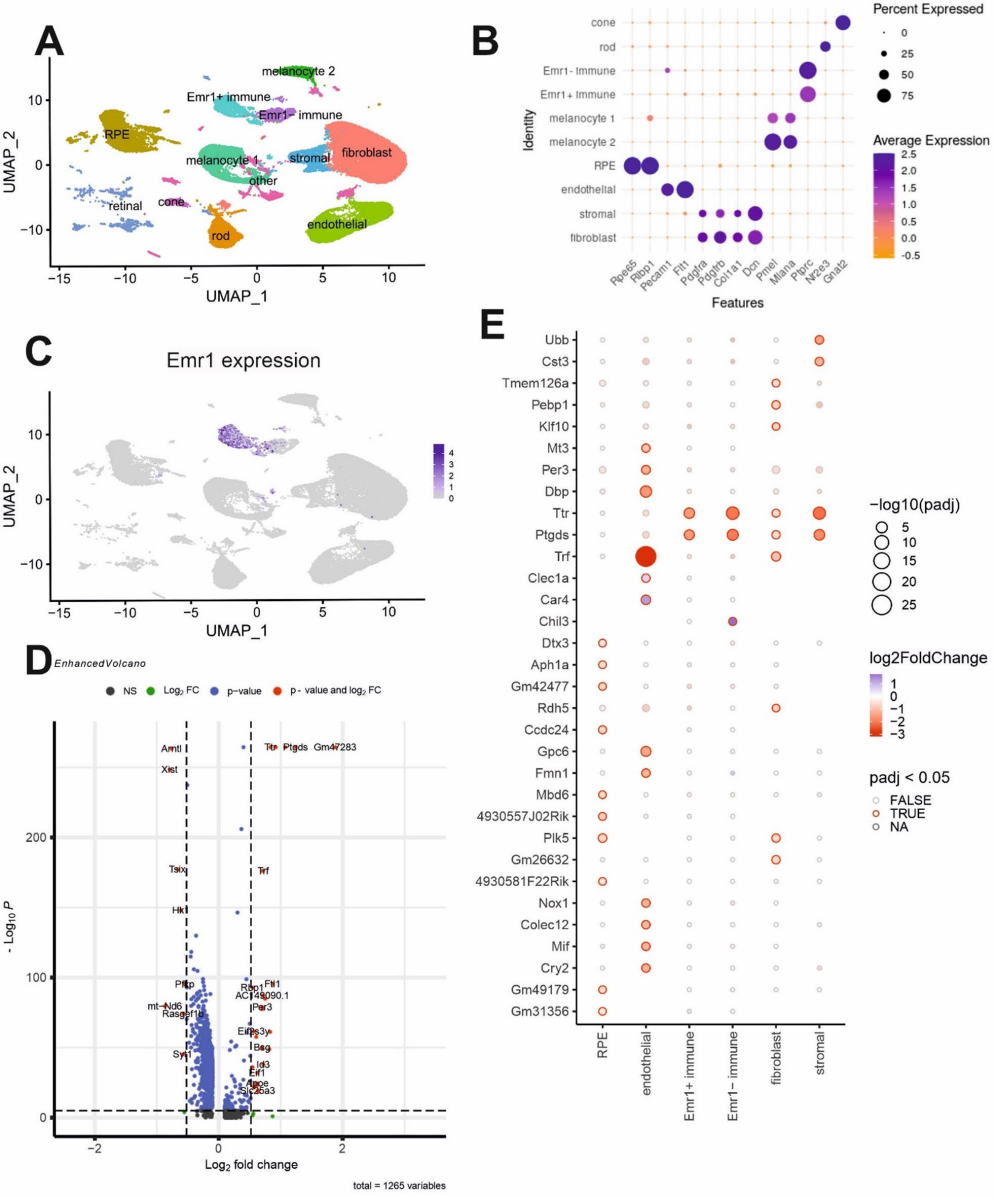
Each cell type involved in CNV is affected by muFHR1. snRNAseq of CNV choroids between WT and *muFHR1*^*−/−*^ 14 days after laser rupture (*n* = 2 × 3). Identified cell types (**A**) based on their expression markers (**B**). Emr1 expression within identified clusters. Differentially regulated genes (**D**) divided by analyzed cell types (**E**)

To assess the overall biological impact of muFHR1, single nuclei RNA sequencing (snRNAseq) of wild type (WT) and *muFHR1*^*−/−*^ RPE/choroids was conducted in laser CNV model 14 days after laser. Each group contained three pooled samples of two RPE/choroids. Following implementation of quality control measures, a total of 47764 cells (22908 WT and 24856 *muFHR1*^−/−^) yielded a uniform manifold approximation and projection (UMAP) to generate a plot of 45 clusters which could be further classified into six primary clusters with distinct expression profiles. Based on key markers for different retinal cell types [48, 49] we identified: RPE, immune cells, endothelial cells, melanocytes and fibroblasts/stromal cells (Fig. 9A-B).

Within all identified clusters, only subsets of RPE cells, immune cells and melanocytes expressed Emr1 (Fig. 9C). As we found differences in gene expression in all cell types comparing WT and *muFHR1*^−/−^ mice, muFHR1 affects also indirectly Emr1^−^ cells (Fig. 9E). Among these differentially expressed genes are strongly regulated genes, including those involved in retinoid cycle (e.g. *Ttr*,* Rbp1*), lipid metabolism and eicosanoid synthesis (e.g. *Ptgds* in immune cells, *ApoE*) and iron regulation (e.g. *Trf*,* Ftl1* in endothelial cells promoting cell migration(66, 67))(Fig. 9C). Emr1^+^ immune cells exhibited an immunogenic predisposition characterized by elevated *Chil3* expression encoding *Ym1* - a regulator of alternative immune activation in mice. Apart from *Chil3*, there was minimal differential gene expression between the mouse genotypes.

**Fig. 10:**
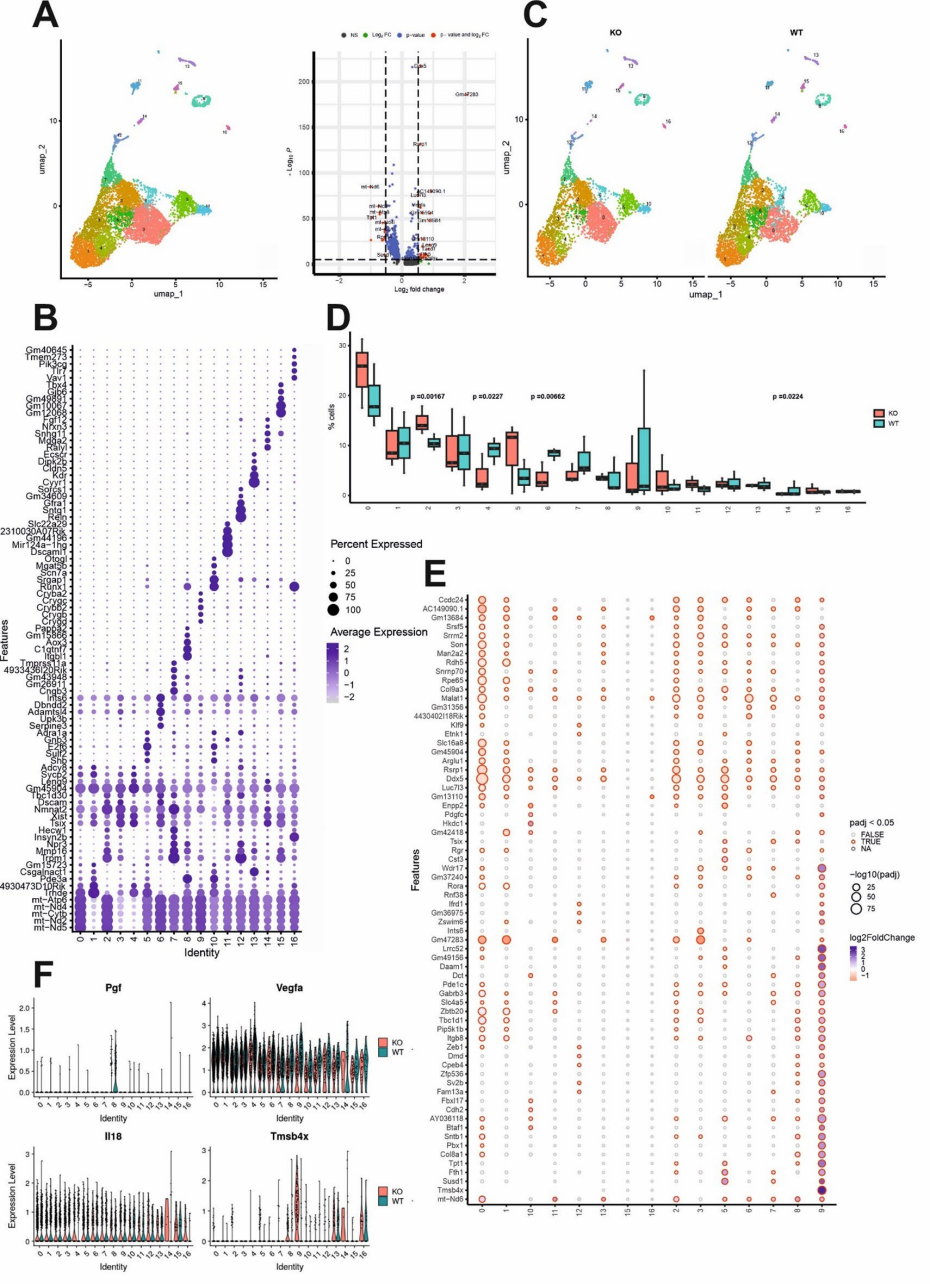
muFHR1 affects RPE subclusters in different ways. Differential gene expression within RPE subtypes (**A**) differentiated by markers (**B**). Genotype-dependent differences (**C**) are observed in cell number (**D**) and gene expression (**E**). MuFHR1 differential expression of genes involved angiogenesis and immune response (**F**)

Given the RPEs’ central role in the pathology of AMD, our research focused on the impact of muFHR1 on RPE cell clusters. Recent publications have demonstrated the presence of distinct sub-specialization within RPE clusters [48, 68–73]. The observation that only individual RPE cells show co-localization of muFHR1/Emr1 in immunohistochemistry prompted us to perform an analysis more in depth on cells with high *Rpe65* expression. We identified a large group of differently expressed genes that identified 16 distinct sub-clusters within *Rpe65*^+^ cells (Fig. 10A and B). The interpretation of their signature expression and the elucidation of their physiological and pathological functions require further investigation (Fig. 10B). In the next step, we have analyzed the effect of muFHR1 presence on these 16 RPE clusters (Fig. 10C). One evident genotype-dependent distinction originates from cell density inside the clusters (Fig. 10D): clusters 0, 2, 5, 11 showed increased density in *muFHR1*^−/−^ mice; clusters 4, 6, 7 and 14 decreased in density in *muFHR*^−/−^

Differential gene expression inside individual RPE subclusters revealed much stronger muFHR1 effects (Fig. 10E) than in the RPE as a complete cluster. Concerning the neovascular environment, the RPE cell subclusters appeared as a major source of *Vegfa*, which was only moderately regulated by muFHR1 (Fig. 10F). In contrast, *Pgf* was exclusively expressed in subcluster 8 and its expression was highly controlled by muFHR1 (Fig. 10F). Further differentially regulated genes within specific clusters between WT and *muFHR1*^−/−^ included *Il18* and *Tmsbx4* (regulator of NF-kB) (Fig. 10F) [71].

## Discussion

The genetic deficiency of the genes *CFHR1* and *CFHR3* has been reported to reduce the risk of developing AMD [29]. Here we explore the functional role of the *CFHR1* gene product FHR1, in the pathophysiology of AMD. We identified a FHR1 initiated Ca^2+^-dependent intracellular second messenger pathway and observed FHR1 labeling in human RPE-choroid tissue samples derived from AMD donors. Subsequently to these findings, we identified the murine FHR1 homolog muFHR1 in RPE/choroid flatmounts obtained from mouse models with relevance to both dry (*TRE2* and *Cx3Cr1*^*GFP/GFP*^) and wet AMD (laser-induced CNV). Furthermore, muFHR1 binds both mononuclear phagocytes (MP) and stressed/damaged RPE cells that express Emr1. Interestingly, we observed colocalization of individual RPE cells, Emr1, muFHR1 and MP. Once bound, FHR1 changes gene expression in the RPE in a Ca^2+^-dependent manner. The FHR1-dependent inflammatory role in AMD is reinforced by the observation of reduced cellular inflammation and angiogenic activity seen in *muFHR1*^*−/−*^ mouse following laser-induced CNV. This reduction is presumably due to substantial reduced infiltration of MPs into the retinal space. The actions of muFHR1 affected RPE, endothelial and immune cells, either directly or indirectly, independent of the Emr1 expression as revealed by snRNAseq. We hypothesize that FHR1 attracts and stabilizes the infiltration of MP into the subretinal space and facilitates cellular inflammation at pre-degeneration state.

FHR1 localizes to Bruch’s membrane and stressed RPE cells in donor eye tissue from AMD patients with geographic atrophy. The localization of FHR1 differs from those of other active complement components in AMD tissue indicating a distinct role in the pathology [9–12] We observed punctate labeling of FHR1 basolateral to the RPE and along Bruch’s membrane in AMD patients. Age-matched controls display significantly less prominent labeling with the exception of one control individual which upon imaging had notable hard drusen beneath the RPE accompanied by anti-FHR1 labeling of Bruch’s membrane and the RPE. The binding of FHR1 to the RPE monolayer from non-diagnosed donors suggests that FHR1 binds not only to apoptotic/necrotic cells [38] but already to stressed cells. Serum levels of FHR1 did not significantly differ between AMD patients and age-matched controls but differed from those of young healthy donors [74] indicating that circulating FHR1 levels increase not only with disease, but also with age.

Although the RPE expresses variety of complement proteins, it lacks the FHR1 expression. However, RPE cells bind FHR1 at a degenerative stage. Previous work [37] identified oxidized surfaces such as oxLDL as a major binding site of FHR1. FHR1 is predominantly expressed in the liver and released into the circulation, so that FHR1 enters the subretinal space from the bloodstream alone or via MPs. Once in the subretinal space, FHR1 acts locally in response to changes in the choroid, Bruch’s membrane and RPE.

In a mouse model exhibiting features of dry AMD, *Cx3Cr1*^*GFP/GFP*^, muFHR1 accumulates at the RPE level prior to any photoreceptor damage. This observation is consistent with the finding that FHR1 accumulates in human specimen with disease, as well as age-dependent cellular changes. A similar pattern was observed in mice with CNV, where muFHR1 bound to RPE cells more distant from the lasered damaged area. Even though our observation focused on muFHR1 apical accumulation, we cannot exclude the possibility of basolateral localization as demonstrated in human samples. Additionally, these binding activities suggest the presence of distinct RPE phenotypes, as stressed, but structurally intact cells express Emr1 and attract muFHR1. This phenomenon, which has not yet been described, could potentially serve as an indicator of RPE cells’ predisposition to its’ degeneration.

The study by Irmscher et al.[38] revealed a pivotal role of FHR1/EMR2 binding in monocytes that could be translated to RPE cells in the context of AMD. In the mouse model, Emr1 expression facilitated interaction between RPE and MP via muFHR1. As such, the binding of muFHR1 to Emr1 expressing RPE cells is of significant interest providing an additional complementary mechanism that impairs the RPE’s barrier function [18].In this way FHR1 potentially enables the infiltration of MP into an otherwise immune-privilege area. Moreover, MPs binding to FHR1 immobilized on the surface of the stressed/degenerated RPE would activate MP’s pro-inflammatory phenotype via EMR2 [38].

Compared to the WT animals, the absence of muFHR1 in laser-induced CNV results in a decrease by more than 50% in number of invading MP, with subsequent less severe scar phenotype, reduced cellular inflammation and reduced angiogenic activity. MuFHR1 deletion reduced the inflammatory activation, which consequently halted sub-retinal inflammatory signals. This correlation between subretinal MPs and severity of neovascular phenotype aligns with previous reports [39, 40, 61–65]. Deficiency of muFHR1 does not result in alterations of the physiological state, a phenomenon that has also been observed in individuals carrying the *CFHR1/CFHR3* deletion [51]. However, when acute inflammation such as sepsis was induced, animals exhibited increased sensitivity [42]. Given the vitality of *muFHR1*^*−/−*^ mice, we hypothesize that muFHR1/Emr1 interaction promotes cell-cell contact only in disease.

Among the differentially regulated genes in the absence of muFHR1, we found *Ttr*,* Trf*,* Ptgds*,* ApoE*; genes known to be differentially regulated in human AMD condition [75]. MuFHR1 alters gene expression in every identified cell type: RPE, immune cells, endothelial cells, and fibroblast/stromal cells independently of their Emr1 expression. Only RPE and immune cells express Emr1 (as confirmed with snRNAseq and immunohistochemistry), and therefore their expression is directly guided via muFHR1/Emr1 interaction. Emr1^−^ cells, which include endothelial cells and fibroblasts, indirectly modify their gene expression, in response to Emr1^+^ cells. The hypothesis is that *muFHR1* deficiency downregulates the inflammatory environment, thereby weakening the signal that adjusts the cell response. It is evident, once more, that the muFHR1 contribution to AMD is intricate, a consequence of its comprehensive impact MP and subsequently on all cell types, irrespective of Emr1 expression.

Concentrating on the RPE population, playing a central role in AMD, we confirmed 16 differential RPE sub-clusters. Despite current efforts to characterize the subtypes of RPE cells and their functional properties their complete profile requires further in-depth investigation [39, 40, 61–65]. However, the evidence suggests that the absence of muFHR1 results in a milder neovascular phenotype. Thus, muFHR1-affected clusters of the RPE subtypes promote pathogenesis, because the sub-clusters vary in numbers and could drive the phenotype, although changes in the RPE gene expression are rather subtle indicating a strong initial response and a strong immune cell effect. The subtle change in *Vegfa* expression across all RPE subclusters indicate that muFHR1 modulates angiogenesis via MPs, and not RPE. In contrast, the *Pgf* expression, which is restricted to a single RPE subcluster is highly sensitive to muFHR1, underscoring another effect by MPs regulating angiogenesis in AMD [61].

Our research revealed properties of FHR1 in AMD pathology comparable to those in atherosclerosis [38]. The binding of (mu)FHR1 to EMR2/1 does not merely serve only to establish a mechanical anchor that facilitates the accumulation of MPs in areas of tissue degeneration, it also fosters a chronic local inflammation. In the light of our in vitro and in vivo observations, FHR1-EMR2 (muFHR1-Emr1) likely elicits a significant influence on the gene expression in RPE cells via a second messenger signaling cascade. In vitro, the quantification of the Ca^2+^ waveform which varied depending on the presence of immobilized FHR1(iFHR1) [38] validated that iFHR1 elicits an increase in intracellular free Ca^2+^ as a second messenger to augment gene expression of IL1β and IL18. Moreover, snRNAseq showed that *Il18* is predominantly expressed from the RPE cells. Notably, while all sub-clusters of RPE cells express *Il18*, only specific clusters down-regulate *Il18* expression in response to muFHR1, thereby contributing to the specific, subtype-dependent muFHR1 effect. So far, the role of FHR1 was purely understood by its influence onto canonical complement activation by perturbing the interaction between CFH and C3. Ca^2+^ signaling analysis together with snRNAseq data imply a new non-canonical FHR1 dependent pathway in AMD.

**Fig. 11:**
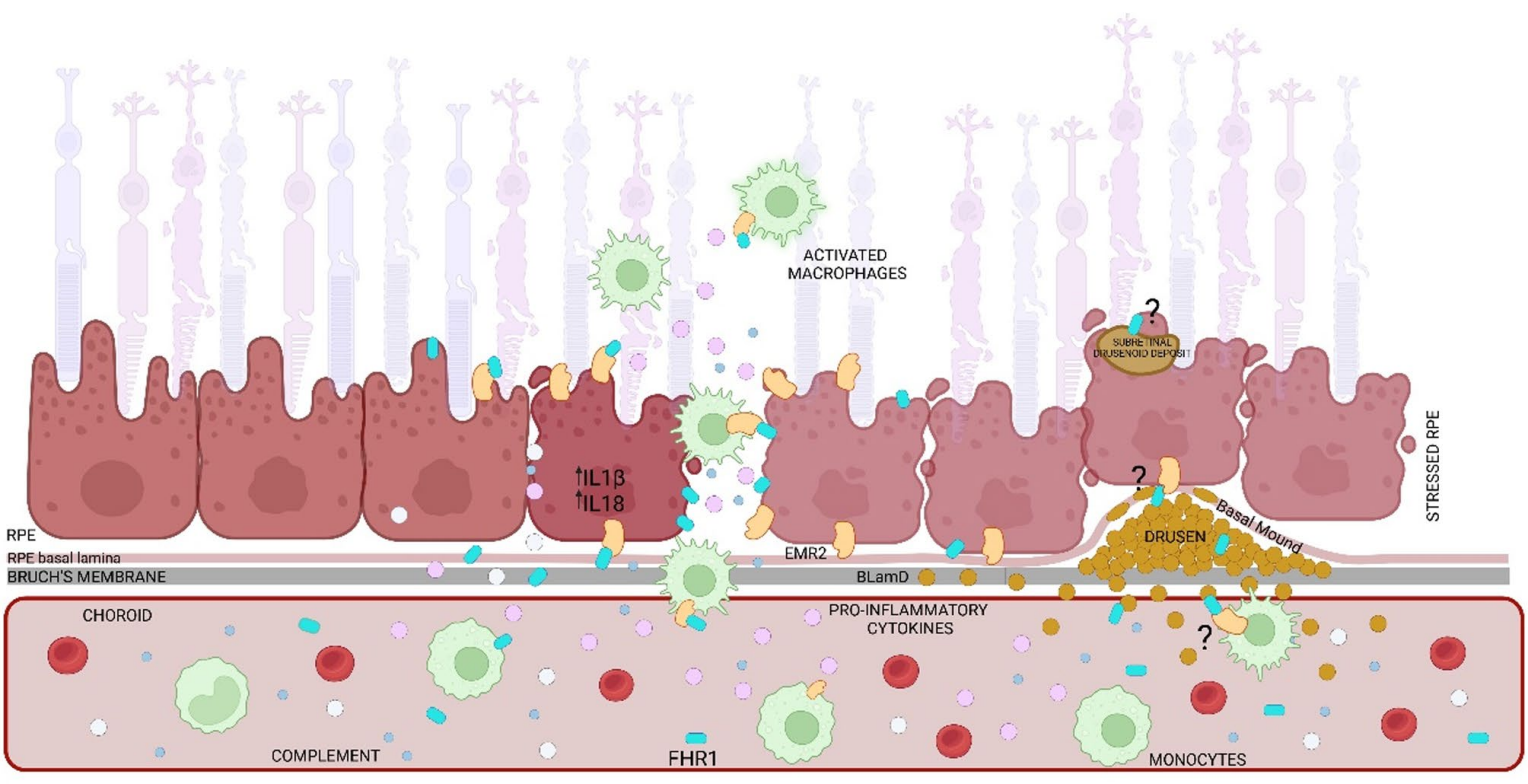
Mechanism-of-action: muFHR1. FHR1 binds to degenerating RPE cells and subsequently: (i) changes the RPE phenotype into a pro-inflammatory one; (ii) allows interaction between RPE and MPs and as such (iii) contributes to the MPs accumulation in the sub-retinal space. FHR1 further over-activates MPs to additionally contribute to the parainflammation. We further hypothesize that FHR1 binds to cholesterol and oxidized lipids within drusen (hallmark of AMD) and subretinal drusenoid deposits to further aggravate inflammation in AMD. Created in BioRender. Sekulic, A. (2025) https://BioRender.com/4oa3kf3

As demonstrated by Irmscher et al. [38], iFHR1 activates monocytes’ inflammasome NF-kB which could serve also as a possible pathway in the RPE. MuFHR1 silences Tmsb4x (cytoplasmic sequestering of NF-kB [71] in the RPE which provides a link between FHR1 and NF-kB in these cells too. Further investigation is necessary to determine its role in the AMD patho-mechanism. In summary (Fig. 11), our findings indicate a dominant role of FHR1 in the patho-mechanism of AMD: (i) FHR1 contributes to MP accumulation in the retina; (ii) EMR2 receptor activation through FHR1 significantly contributes to para-inflammation; (iii) already stressed cells enter the FHR1 signaling prior to manifestation of initial clinical symptoms; (iv) FHR1 facilitates physical interaction between MP and RPE and instigates intracellular second-messenger signaling with the potential to modify RPE’s functional phenotype.

To clarify the mechanism by which FHR1 selectively binds to and possibly identifies RPE cells predisposed to degeneration requires further research. Detailed investigation of RPE subtypes, their functionality and age/disease-progression is necessary to fully understand RPE role. Additionally, while our focus was on non-canonical function of FHR1, we cannot exclude the role of canonical pathway: FHR1 competes with CFH to bind C3b and promote complement activation. Given its role in inflammation (both canonical and non-canonical), FHR1 may contribute to the limited success of existing AMD therapies by providing an alternative pathological mechanism. However, investigating FHR1 in early AMD before clinical symptoms arise is limited to mice models that do not fully recapitulate the complexity of human AMD. Furthermore, our research only provides an initial insight into the role of AMD pathogenesis which needs additional confirmation on a larger scale.

FHR1 was recently described as an important factor igniting cellular inflammation in atherosclerotic plaques [37, 38]. Our data imply a comparable mechanism of FHR1 in cellular inflammation in AMD (Fig. 11). AMD results from aging processes acting at the RPE integrity over decades. These processes include chronic exposure to photooxidative stress, causing oxidative damage to large variety of vulnerable molecules leading the formation of lipofuscin [76] and oxidized lipids among them oxLDL [77]. In early AMD, there is a degeneration or even a loss of RPE cells, going along with chronic cellular inflammation including MP invasion and complement activation, the latter fostered by polymorphic CFH. At this stage FHR1 might accelerate this process through binding competition between CFH and FHR1 for C3b. In an advanced state, MPs reach the subretinal space through a FHR1-dependent mechanism. FHR1 becomes more and more immobilized on oxLDL surfaces (non-EMR2 process) and by binding to EMR2 expressing cells: the RPE and MPs (Figs. 1, 2, 3, 4 and 5). The EMR2/FHR1 binding renders MPs to become active [78] and the weakened RPE immune barrier permits the invasion of MPs into the subretinal space with RPE cells expressing abundantly EMR2. The RPE does not express FHR1 but the invading MP carry FHR1 into the subretinal space (Figs. 3 and 4) and stabilize the cellular inflammation in an area that normally represents an immune privileged space (Figs. 3, 4 and 8). FHR1 binding to EMR2 activates a Ca^2+^-dependent signaling cascade in the RPE that changes gene expression towards a more pro-inflammatory phenotype (Figs. 6, 7, 9 and 10). Thus, we hypothesize that FHR1 acts on two cell types, RPE cells and MP, which first increases and then stabilizes the cellular inflammatory network in degenerative stages, such as AMD and atherosclerosis. This may explain why naturally occurring FHR1 deficiency in humans reduces the risk for AMD but is unlikely to prevent AMD.

## Electronic supplementary material

Below is the link to the electronic supplementary material.

Supplementary Material 1

## Data Availability

The data generated in this study have been deposited at the Zenedo repository here: 10.5281/zenodo.14961318.

## Abbreviations

AMD: Age-related macular degeneration
RPE: Retinal pigment epithelium
MP: Mononuclear phagocytes
FHR1: Factor H-related protein 1
EMR2: EGF-like module containing mucin-like hormone receptor 2
CFH: Complement factor H
NHS: Natural human serum
VEGF: Vascular endothelial growth factor
KO: Knock out
FA: Fluorescence angiography
IntDen: Integrated density
EDC: 1-Ethyl-3-(3-dimethylaminopropyl)carbodiimide)
NHS: N-hydroxysuccinimide
MES: 2-Morpholinoethanesulphonic acid
